# Welcome to the jangle (fallacy): The case of statistics and mathematics anxieties

**DOI:** 10.1111/bjep.70119

**Published:** 2026-08-16

**Authors:** Jenny Terry, Charlie Lea, Andy P. Field

**Affiliations:** ^1^ School of Psychology, Faculty of Science, Engineering and Mathematics University of Sussex Brighton UK; ^2^ School of Humanities and Social Science University of Brighton Brighton UK

**Keywords:** construct validity, jangle fallacy, mathematics anxiety, statistics anxiety

## Abstract

**Background:**

Statistics‐related anxiety is a common barrier to learning in higher education, particularly for psychology and other non‐specialist students. High prevalence rates are concerning because statistical literacy is integral to academic success, employability and informed citizenship. Clarifying the nature of statistics anxiety is central to understanding it, yet it remains unclear whether statistics anxiety is genuinely distinct from mathematics anxiety or simply another manifestation of the same construct.

**Aims:**

We examined the degree of overlap (jangle fallacy) between the Statistics Anxiety Rating Scale (STARS) and the Revised Mathematics Anxiety Rating Scale (R‐MARS).

**Samples:**

Two cohorts of UK undergraduate psychology students participated: Study 1 (*n* = 489) and Study 2 (*n* = 245).

**Methods:**

Each study combined a cross‐sectional survey with a between‐participants experiment. Surveys included the STARS, R‐MARS, parallel forms to control for scale content and measures of trait and state anxiety. In the experiment, participants were randomly assigned to complete either a statistics or mathematics multiple‐choice test, with state anxiety measured before and after.

**Results:**

Results pointed to overlap across all analyses and samples. Scales were highly correlated, items did not separate into distinct domains, individuals rarely reported one without the other, and state anxiety increased similarly across test types.

**Conclusions:**

Findings show that statistics anxiety, as measured by the STARS, is not distinct from mathematics anxiety, as measured by the R‐MARS. Recognizing statistics anxiety as part of the broader mathematics anxiety construct can streamline measurement, clarify theory and support more effective interventions for reducing quantitative anxieties in education.

## INTRODUCTION

Educational anxieties are among the most reported barriers to learning and achievement in university‐level education. Constructs such as test anxiety, mathematics anxiety and statistics anxiety are frequently cited as reasons for avoidance, poor engagement and underperformance in quantitatively demanding components of undergraduate curricula (Chew & Dillon, 2014; Hembree, 1990; Zeidner, 1991). In psychology and related social science degrees in particular, statistics is often regarded as one of the most intimidating subjects (Bourne & Nesbit, 2018). Surveys of psychology undergraduates suggest that more than half of students report anxiety as a primary reason for difficulties learning statistics (Field, 2014) and prevalence estimates indicate that up to 84% experience moderate to high levels of statistics anxiety (Kawsar et al., 2019). These figures are concerning given that statistical literacy is increasingly recognized as a core outcome of many psychology, social science and STEM degrees because of its relevance for academic success, professional competence and active citizenship in a data‐rich world (Gal, 2002).

Given its prevalence and impact, statistics anxiety has attracted sustained research attention for several decades. Yet despite this extensive literature, fundamental questions remain unresolved. We lack a clear understanding of its developmental origins, its influence on students' learning trajectories and—perhaps most crucially—the precise nature of the construct itself. One persistent issue is whether statistics anxiety is genuinely distinct from mathematics anxiety or simply an expression of the same underlying construct in a different context (Terry & Field, 2024). If the latter is true, then the ‘statistics anxiety’ literature may be an example of a jangle fallacy (Kelley, 1927), in which a construct (and/or its measures) is given more than one name.

Such fallacies are more than theoretical curiosities. They risk fragmenting literatures into parallel silos (Block, 1995), slowing theoretical progress, duplicating interventions and introducing statistical artefacts such as multicollinearity when overlapping scales are used in the same predictive models (Mason & Perreault, 1991). Clarifying the distinctiveness between educational anxieties, such as mathematics and statistics, is therefore not only a psychometric concern but a central question for educational psychology with direct implications for pedagogy.

### Is statistics anxiety distinct from mathematics anxiety?

To clarify whether statistics anxiety is distinct from mathematics anxiety it is necessary to examine the different sources of validity evidence recognized in contemporary testing standards (American Educational Research Association et al., 2014).

#### Evidence based on test content

Attempts to distinguish statistics anxiety from mathematics anxiety have generally rested on subtle definitional differences. Cruise et al. (1985, p. 92) described statistics anxiety as ‘the feelings of anxiety encountered when taking a statistics course or doing statistical analyses; that is gathering, processing and interpreting data’, whereas Richardson and Suinn (1972, p. 551) defined mathematics anxiety as ‘feelings of tension and anxiety that interfere with the manipulation of numbers and the solving of mathematical problems’. These descriptions share substantial conceptual territory: both describe affective responses to quantitative manipulation and problem‐solving, with the supposed distinction hinging only on whether the context is labelled ‘statistics’ or ‘mathematics’. From a test content perspective, the overlap in foundational definitions undermines the basis for unique scale content. That is, if the conceptual domains are not clearly distinct, then the items derived from them cannot be expected to represent separate constructs.

#### Evidence based on relations to other variables

A central source of validity evidence concerns how test scores relate to other variables in theoretically expected ways (American Educational Research Association et al., 2014). In this case, such evidence falls into two categories: the direct association between statistics and mathematics anxieties themselves and the extent to which both show parallel relations with other constructs within their nomological networks.

Evidence for direct association comes from correlations between composite scores on statistics and mathematics anxiety measures. Early studies, which relied on bespoke or single‐item measures, typically reported correlations ranging from moderate to strong. Zeidner (1991), for example, found a correlation of *r* = .41 between a statistics anxiety scale and a single‐item measure of mathematics anxiety, with additional correlations of *r* = .28–.36 between mathematics anxiety and subcomponents of statistics anxiety. Birenbaum and Eylath (1994) reported *r* = .54 between their ten‐item mathematics anxiety scale and a single‐item statistics anxiety measure. Tremblay et al. (2000) reported a stronger association of *r* = .81 between a ten‐item ‘numerical anxiety’ and a ten‐item statistics anxiety, suggesting almost complete overlap.

More recent studies using multidimensional validated scales have generally pointed to overlap. Primi and Chiesi (2018) reported *r* = .61 between the Statistics Anxiety Rating Scale (STARS; Cruise et al., 1985) and the Abbreviated Mathematics Anxiety Scale (AMAS; Hopko et al., 2003). Nasser (2004) found that correlations between the STARS, the Revised Mathematics Anxiety Rating Scale (R‐MARS; Plake & Parker, 1982) and the Survey of Attitudes Toward Statistics (SATS; Schau et al., 1995) were so high that the STARS had to be removed from their structural equation models due to collinearity. Gibeau et al. (2023) likewise reported *r* = .79 between the Statistics Anxiety Scale (Vigil‐Colet et al., 2008, adapted from the STARS) and both the AMAS and a single‐item measure of mathematics anxiety.

Beyond these direct associations, evidence of nomological networks considers whether constructs relate to other variables in empirically or theoretically similar ways. Here, too, statistics and mathematics anxiety appear difficult to separate. Both anxieties show similar relations with attitudes towards statistics, willingness and/or motivation to study statistics and prior statistics grades (Birenbaum & Eylath, 1994; Tremblay et al., 2000; Zeidner, 1991). Consistent with this, Grimes and Gardner (2023) identified fifteen categories of antecedents for statistics anxiety, eleven of which were identical to antecedents of mathematics anxiety. The remaining four—including teacher support and negative classroom experiences—have also been linked to mathematics anxiety in other work (e.g., Luttenberger et al., 2018). Developmental pathways and associations with relevant concepts therefore appear shared, with statistics anxiety perhaps best understood as a context‐specific manifestation of a broader mathematics‐related anxiety.

Theoretical accounts also point in the same direction. Frameworks of statistics anxiety have typically been adapted from models of mathematics anxiety with both constructs theorized to relate to other constructs (e.g., academic performance, self‐efficacy, persistence) in similar ways (Terry & Field, 2024).

#### Evidence based on internal structure

Relying on associations as an indicator of overlap is problematic because two variables can be associated for other reasons, such as the influence of a third variable. Confirmatory factor analytic evidence can provide a more direct test of distinctiveness. We found only one study that took such an approach. Paechter et al. (2017) examined the overlap between the two constructs using CFA of the STARS and R‐MARS (Baloğlu & Zelhart, 2007). Their results indicated the two constructs were very strongly associated (β = .855), suggesting considerable overlap, even though the confirmatory factor analysis supported modelling them as two separate factors. Interpretation of these findings is constrained in several ways. First, the relatively small sample size (*n* = 225) is unlikely to produce stable factors. Second, the STARS and R‐MARS were administered at different time points, introducing potential time‐related confounds (e.g., changes in academic context, experience or affect). Third, the inclusion of structural paths alongside measurement models blurred evidence about factor structure with evidence about predictive relations. The present study directly addresses these limitations by administering both measures concurrently and evaluating their relationship within a purely measurement‐focused framework.

Yet another challenge when comparing measures of statistics and mathematics anxiety, is that they differ in their coverage of educational experiences. For example, the STARS includes a Fear of Asking for Help subscale, absent from the R‐MARS. More broadly, both measures contain items referring to similar educational activities that differ only in disciplinary framing (e.g., studying for a mathematics vs. a statistics examination), alongside items that refer to somewhat different activities (e.g., interpreting statistical results in journal articles versus solving mathematical calculations). Such non‐shared elements introduce unique variance that can create an illusion of distinctiveness.

Apparent distinctions between statistics and mathematics anxiety may, therefore, reflect two different possibilities. The first is that anxieties are primarily domain‐driven, such that responses cluster according to disciplinary labels (i.e., statistics versus mathematics), regardless of the specific educational activity involved. For example, anxiety about walking into a statistics test would be more closely related to anxiety about interpreting statistical results than to anxiety about walking into a mathematics test. The second possibility is that anxieties are more task‐driven, clustering around shared educational experiences or cognitive activities—such as tests, interpretation, calculation, classroom participation or asking for help—irrespective of whether these occur in mathematics or statistics contexts. Under this account, anxiety about walking into a statistics test would be more closely related to anxiety about walking into a mathematics test than to other statistics‐specific activities. Distinguishing between these possibilities is central to determining whether statistics anxiety represents a distinct construct rather than a context‐specific manifestation of broader mathematics‐related anxieties. The present study design allowed us to test whether anxieties clustered primarily by disciplinary domain or by shared educational tasks and experiences.

### The present research

The inconsistencies and limitations in previous work mean we cannot be sure whether statistics anxiety scales capture a construct that is genuinely distinct from mathematics anxiety scales or instead reflect a manifestation of the same phenomenon. A clearer understanding of this relationship has implications for educational theory and practice, including how anxiety‐related barriers to learning are identified, measured and targeted.

We adopted a multi‐pronged approach that addressed previous limitations. We focused on the widely used Statistics Anxiety Rating Scale (STARS; Cruise et al., 1985) and the Revised Mathematics Anxiety Rating Scale (R‐MARS; Baloğlu & Zelhart, 2007) to enable direct comparison with Paechter et al.'s (2017) study. These instruments also have items common to other statistics (Faraci & Malluzzo, 2024) and mathematics anxiety scales (Luttenberger et al., 2018), thereby broadening the generalizability of our findings.

As the two scales differ in their subscale structure and item content, observed differences could reflect measurement artefacts (e.g., subscales unique to one scale) rather than genuine construct distinctiveness. To address this, we created parallel versions of each scale by adapting item wording to refer to the alternate domain (i.e., statistics items rewritten for mathematics contexts and vice versa). This allowed us to compare responses to matched items across domains while holding broader educational tasks and experiences relatively constant. Consequently, if apparent distinctions between statistics anxiety and mathematics anxiety diminished once item framing and subscale composition were aligned, this would suggest that previously observed differences were driven at least partly by measurement characteristics rather than genuinely distinct anxieties. Conversely, persistent separation despite these adaptations would provide stronger evidence for domain‐specific anxiety. More broadly, this design allowed us to test whether any apparent unique variance reflected genuinely domain‐specific anxiety or instead arose from anxieties tied to shared educational tasks and experiences (e.g., tests, interpretation, calculation or classroom participation) that were unevenly represented across the original measures.

In addition to examining associations between the scales, we used several complementary analytical approaches to evaluate the distinctiveness of statistics and mathematics anxiety across two independent cohorts of UK psychology undergraduates. First, factor analytic approaches were used to examine structural overlap between the STARS, R‐MARS and their parallel versions. Specifically, exploratory factor analyses tested whether items clustered according to disciplinary domain (statistics versus mathematics) or according to shared educational tasks and experiences, while confirmatory factor analyses evaluated the resulting structures. We reasoned that substantial cross‐domain clustering would suggest that the scales represent a broader shared construct rather than distinct anxieties. Second, latent profile analyses examined whether individuals tended to show comparable levels of statistics and mathematics anxiety or whether dissociable profiles emerged (e.g., high statistics anxiety with low mathematics anxiety), which would support construct distinctiveness. Third, multilevel modelling of experimental data tested whether statistics anxiety and mathematics anxiety showed domain‐specific predictive validity. Participants completed either a mathematics or statistics test modelled on assessment formats commonly encountered by students, providing discipline‐specific evaluative contexts in which we could examine whether state anxiety responses varied according to congruence between domain‐specific anxiety and task domain. Specifically, if the statistics and mathematics anxiety constructs are meaningfully distinct, statistics anxiety should be more strongly associated with state anxiety in the statistics test condition, whereas mathematics anxiety should be more strongly associated with responses in the mathematics test condition. Conversely, if both anxieties largely reflect the same underlying construct, their associations with state anxiety should be similar regardless of the type of test.

Together, these complementary approaches provide one of the most comprehensive tests of the distinctiveness of statistics and mathematics anxieties to date.

## METHODS

### Participants

Data were collected from two universities in the South‐East of England (see Table 1 for sample characteristics, exclusions and demographics). At both sites, all undergraduate psychology students who had at least begun their first introductory statistics module were invited to participate via the participant pool system in exchange for course credits (Study 1) or prize draw entry points (Study 2). Data collection took place for Study 1 between March and April 2020, and for Study 2 between October 2020 and June 2021.

**TABLE 1:** Sample characteristics, exclusions and demographics per study.

Sample property	Study 1	Study 2
Undergraduate psychology population	~1500	~800
Course entry requirements	AAB–ABB A‐Level; GCSE Maths ≥5/B	BBB–BCC A‐Level; GCSE Maths & English ≥4/C
Initial *N*	588	268
Exclusions: No consent	3	4
Exclusions: Empty responses	65	12
Exclusions: Duplicate responses	4	2
Exclusions: Speedy responders	27	5
Final *N*	489	245
Gender: Women	374 (76.5%)	197 (80.4%)
Gender: Men	92 (18.8%)	36 (14.7%)
Gender: Non‐binary	3 (.6%)	8 (3.3%)
Age (M, SD, range)	20.47 (2.85), 17–49	21.72 (4.40), 18–43
Ethnicity: White	76.5%	79.2%
Year of study: Year 1	44.0%	41.6%
Year of study: Year 2	49.9%	52.2%
Learning differences: None	84.3%	76.3%
Learning differences: Dyscalculia	.2% (*n* = 1)	1.2% (*n* = 3)
Mathematics background: ≥GCSE C	99.6%	100%
Mathematics background: A‐Level	27.0%	11.0%

*Note*: GCSE = A qualification typically completed at age 16 in the UK; A‐Level = Advanced Level qualification, typically completed at age 18 and commonly used for university admission. Empty responses = 0% of questions answered; speedy responders = completion rate <4.85 s per 1% progress, MCQ time excluded (see preregistration for more details).

Abbreviations: GCSE, General Certificate of Secondary Education; M, mean; SD, standard deviation.

A minimum target of 300 participants per study was set based on simulation studies suggesting this as an adequate sample size for our factor analytic and latent profile analyses (MacCallum et al., 1994; Tein et al., 2013). Multilevel models were used for effect estimation rather than hypothesis testing, so no a priori power calculations were necessary.

This study was approved (ER/JLT26/5) by the Sciences & Technology Cross‐Schools Research Ethics Committee (C‐REC) at the University of Sussex and adhered to the British Psychological Society's Code of Human Research Ethics (British Psychological Society [BPS] Ethics Board, 2018).

### Design

This project employed two complementary designs across both studies:

1. A cross‐sectional self‐report survey assessed statistics and mathematics anxieties with four measures for use in factor analytic and latent profile analyses (see Data Analysis)
2. A between‐participants experiment in which participants were assigned randomly to complete either a statistics or a mathematics multiple‐choice test, with state anxiety measured before and immediately after, the latter assessment referring to participants' anxiety during test completion

### Materials

All materials are available on the project's OSF page (https://osf.io/8jk5v).

#### Measures

##### Statistics anxiety

The three anxiety subscales of the Statistics Anxiety Rating Scale (STARS; Cruise et al., 1985) were used (dropping the three statistics attitudes subscales (Hanna et al., 2008; Papousek et al., 2012)): Test and Class Anxiety (8 items), Interpretation Anxiety (11 items) and Fear of Asking for Help (4 items). Items describe situations involving statistics, rated from 1 (‘no anxiety’) to 5 (‘very much anxiety’). Several items were updated to reflect contemporary language (e.g., ‘statistical output’ instead of ‘printout’). Reliability was excellent (Study 1: ω_t_ = .94 [.94, .95]; Study 2: ω_t_ = .93 [.92, .95]).

##### Mathematics anxiety

Mathematics anxiety was measured with the Revised Mathematics Anxiety Rating Scale (R‐MARS; Baloğlu & Zelhart, 2007), comprising three subscales: Mathematics Test Anxiety (15 items), Numerical Task Anxiety (5 items) and Mathematics Course Anxiety (5 items). Items describe situations such as taking a maths exam or solving arithmetic problems, rated from 1 to 5. US‐specific items were modified for UK equivalence (e.g., ‘college entrance exam’ was changed to ‘university entrance exam’). Reliability was excellent (Study 1: ω_t_ = .94 [.94, .95]; Study 2: ω_t_ = .94 [.93, .95]).

##### Parallel scales

To test whether apparent differences between statistics and mathematics anxiety reflected scale content rather than construct distinctiveness, we created adapted (‘parallel’) forms of each measure: a mathematics version of the STARS (STARS‐M) and a statistics version of the R‐MARS (R‐MARS‐S). In these versions, items were systematically rewritten to refer to the alternate domain while preserving their original structure and response format (e.g., ‘Doing the coursework for a statistics course’ was revised to ‘Doing the coursework for a mathematics course’; ‘Walking into a mathematics class’ was revised to ‘Walking into a statistics class’). Items that were not clearly specific to either domain were not adapted and were retained in their original form (three STARS items and one R‐MARS item; see (Terry et al., 2023) for more details). Reliability was excellent (Study 1: ω_t_ = .94–.94; Study 2: ω_t_ = .93–.94).

##### Trait and state anxiety

The State–Trait Inventory for Cognitive and Somatic Anxiety (STICSA; Ree et al., 2008). The STICSA contains comparable trait and state versions of 21 items each, covering somatic and cognitive symptoms (e.g., ‘My heart beats fast’/‘I cannot concentrate without irrelevant thoughts intruding’), rated 1 (‘not at all’) to 4 (‘very much so’). Minor wording edits were made (e.g., present tense in state items). The pre‐experimental manipulation (herein, ‘pre‐test’) version of the state anxiety scale instructed participants to ‘read each statement carefully and select the number which best indicates how you feel right now, at this very moment, even if this is not how you usually feel’, while the post‐experimental manipulation (herein, ‘in‐test’) version instructed participants to ‘read each statement carefully and select the number which best indicates how you felt whilst answering the multiple choice questions, even if this is not how you usually feel’. Reliability was excellent for trait anxiety and both pre‐test and in‐test state anxiety (Study 1: ω_t_ = .94–.95; Study 2: ω_t_ = .92–.93).

##### Prior education

Participants reported their highest completed level of mathematics and statistics education (including current degree modules) and the grades achieved.

##### Demographics

Participants provided age, gender identity, ethnicity and whether they had a diagnosed specific learning difference (e.g., dyslexia, dyscalculia).

#### Experimental manipulation

##### Multiple‐choice questions (MCQs)

The experimental manipulation consisted of randomly assigning participants to one of two conditions, each involving a five‐item multiple‐choice questionnaire (MCQ). In the statistics condition, participants completed a statistics‐focused MCQ, whereas in the mathematics condition, they completed a mathematics‐focused MCQ. The two versions were designed to be equivalent in structure and content while differing in disciplinary framing. Both versions of the task covered the same five concepts (mean, standard deviation, confidence intervals, raw β coefficients, standard error) using identical numerical values. The mathematics version required purely numerical calculation, whereas the statistics version embedded the same numbers in a research context and required interpretation. For example, the maths item asked, ‘What is the mean of the following set of numbers?’, whereas the statistics item asked students to interpret a mean rating of 61 in a survey context. The MCQs are available on the project's OSF page (https://osf.io/h6zgx).

Descriptive indicators of MCQ performance suggested that the statistics MCQs were generally lower than the mathematics MCQs, particularly in Study 2 (see Appendix S1, Section A).

### Procedure

Participants completed the study remotely on PCs or mobile devices via a Qualtrics survey (Qualtrics, 2020) accessed through their university's participant pool system. Before beginning the survey, participants were provided with an information sheet and gave consent to participate. The information sheet explained that participants would complete a 5‐question multiple‐choice test, but did not specify whether this would involve mathematics or statistics content. Once they started the survey, participants first completed all four statistics and mathematics anxiety scales, with both scales and items randomized. This was followed by the trait anxiety scale and then the state anxiety scale, each randomized at the item level. Participants were then randomly assigned to either the statistics or mathematics version of the MCQ test. Response options were randomized, but the question order was fixed. Instructions prohibited calculator use (although this could not be verified, owing to the remote design). A countdown timer was displayed, and the survey advanced automatically once time expired. Immediately after the MCQ test, participants completed the state anxiety scale again (items randomized) based upon how anxious they felt during the test, followed by questions about prior mathematics and statistics education and demographic information. Finally,^1^ participants were debriefed and thanked. The study took approximately 15 min.

### Data analysis

Analyses were preregistered early in the first author's PhD. Some analytical details deviate from the preregistration, reflecting advances in their methodological understanding. The preregistered analyses were also run as sensitivity checks and produced no substantive differences in our conclusions. Preregistrations, deviations and sensitivity checks are documented on the OSF (https://osf.io/f7rqn).

Study 1 began with confirmatory factor analyses (CFA) of the original STARS and R‐MARS structures as a baseline check to ensure the measures worked as intended in our sample. We then calculated Pearson correlations among the composite scores of the STARS, R‐MARS and their modified forms (STARS‐M, R‐MARS‐S), followed by a set of core analyses: (1) exploratory factor analyses (EFA) to test whether items clustered by construct or domain; (2) latent profile analyses (LPA) of composite scores to examine whether individuals showed dissociated profiles of statistics versus mathematics anxiety; and (3) multilevel models of the experimental data to test whether the two measures predicted state anxiety specifically within their respective domains.

Study 2 repeated the Study 1 analyses for corroboration, each with the same specifications. The only exception was that rather than repeating the EFA, the factor structure identified in the Study 1 EFA was tested using CFA.

## RESULTS

Data analyses were conducted using R 4.3.3 (R Core Team, 2025) via RStudio 2024.04.1 + 748 (Posit Team, 2025). Anonymized data (https://osf.io/svakx) and R code (https://osf.io/qwgmz) are available via the project's OSF pages. Unless otherwise specified below, data exclusions were limited to those described in the Participants section. Results from Studies 1 and 2 are reported together, organized by analysis type.

### Descriptive statistics

Table 2 shows the composite‐ and subscale‐level descriptive statistics for Studies 1 and 2. Average statistics anxiety (STARS and R‐MARS‐S) ratings were higher than mathematics anxiety (R‐MARS and STARS‐M) ratings at both universities, and Study 2 had higher mean scores than Study 1 on all measures. See (Appendix S1, Section B) for item‐level descriptive statistics.

**TABLE 2:** Descriptive statistics for all composite and subscale variables in the Study 1 and 2 samples.

Variable	Study	M	SD	Min	Max	95% CI
STARS	1	2.94	.79	1.04	4.91	[2.87, 3.01]
	2	3.23	.75	1.43	4.96	[3.14, 3.32]
STARS – Interpretation	1	2.62	.86	1.00	5.00	[2.54, 2.70]
	2	2.93	.88	1.00	5.00	[2.82, 3.04]
STARS – Test & class	1	3.43	.86	1.12	5.00	[3.35, 3.50]
	2	3.75	.76	1.38	5.00	[3.66, 3.85]
STARS – Asking for help	1	2.83	1.13	1.00	5.00	[2.73, 2.93]
	2	3.01	1.13	1.00	5.00	[2.87, 3.15]
STARS‐M	1	2.88	.80	1.00	4.95	[2.81, 2.95]
	2	3.14	.77	1.00	4.85	[3.04, 3.24]
STARS‐M – Interpretation	1	2.47	.85	1.00	5.00	[2.39. 2.54]
	2	2.74	.88	1.00	4.82	[2.62, 2.85]
STARS‐M – Test & class	1	3.29	.90	1.00	5.00	[3.21, 3.37]
	2	3.61	.82	1.00	5.00	[3.50, 3.71]
STARS‐M – Asking for help	1	2.76	1.08	1.00	5.00	[2.67, 2.86]
	2	2.87	1.13	1.00	5.00	[2.73, 3.01]
R‐MARS	1	2.79	.78	1.00	4.85	[2.72, 2.86]
	2	3.05	.78	1.20	4.70	[2.95, 3.15]
R‐MARS – Test	1	3.48	.90	1.00	5.00	[3.40, 3.56]
	2	3.84	.82	1.20	5.00	[3.73, 3.94]
R‐MARS – Course	1	2.25	.90	1.00	5.00	[2.17, 2.33]
	2	2.39	.94	1.00	4.80	[2.27, 2.51]
R‐MARS – Numeric tasks	1	1.93	.86	1.00	4.80	[1.85, 2.00]
	2	2.15	.95	1.00	5.00	[2.03, 2.27]
R‐MARS‐S	1	3.19	.81	1.05	5.00	[3.12, 3.26]
	2	3.56	.75	1.37	5.00	[3.47, 3.65]
R‐MARS‐S – Test	1	3.72	.85	1.10	5.00	[3.64, 3.79]
	2	4.09	.66	1.70	5.00	[4.01, 4.17]
R‐MARS‐S – Course	1	2.42	.91	1.00	5.00	[2.34, 2.50]
	2	2.64	.94	1.00	5.00	[2.52, 2.76]
R‐MARS‐S – Numeric tasks	1	2.54	1.00	1.00	5.00	[2.45, 2.63]
	2	3.02	1.05	1.00	4.80	[2.89, 3.15]
Trait anxiety	1	2.21	.63	1.00	3.90	[2.15, 2.27]
	2	2.49	.64	1.00	4.00	[2.41, 2.57]
State anxiety (pre‐test)	1	1.78	.62	1.00	3.71	[1.72, 1.83]
	2	1.96	.62	1.00	3.71	[1.88, 2.04]
State anxiety (in‐test)	1	1.78	.64	1.00	3.95	[1.72, 1.84]
	2	2.10	.69	1.00	4.00	[2.01, 2.19]

Abbreviations: 95% CI, 95% confidence interval; M, Mean; Max, maximum recorded value; Min, minimum recorded value; SD, standard deviation.

### Correlations

Table 3 presents Pearson correlations at the composite and subscale level of all statistics and mathematics anxiety measures, for both Study 1 (lower triangle) and Study 2 (upper triangle). All composite statistics and mathematics anxiety scores were strongly correlated in both samples (*r* = .68–.85). At the subscale level, correlations were highest for content‐matched subscales (e.g., test‐related with test‐related, course‐related with course‐related), regardless of whether items referred to statistics or mathematics (typically *r* ≈ .66–.92). In contrast, correlations between non‐matched subscales were smaller and more variable (approximately *r* ≈ .26–.59). See Appendix S1, Section C for item‐level correlations.

**TABLE 3:** Pearson correlations (with pairwise deletion) for all composite variables in the Study 1 (lower triangle) and Study 2 (upper triangle) samples.

	1	2	3	4	5	6	7	8	9	10	11	12	13	14	15	16	17	18	19
1 – STARS	–	.92	.87	.70	.83	.81	.67	.61	.68	.58	.68	.56	.82	.72	.76	.66	.45	.36	.50
2 – STARS – Int.	.92	–	.68	.46	.75	.89	.55	.42	.60	.46	.63	.53	.73	.58	.69	.67	.36	.29	.44
3 – STARS – Test/class	.88	.69	–	.53	.72	.61	.74	.46	.69	.68	.61	.48	.81	.82	.69	.58	.42	.32	.45
4 – STARS – Help	.72	.52	.52	–	.59	.38	.41	.84	.42	.33	.44	.35	.47	.43	.50	.32	.40	.32	.37
5 – STARS‐M	.84	.76	.76	.61	–	.86	.88	.73	.82	.73	.77	.66	.71	.63	.70	.54	.51	.40	.50
6 – STARS‐M – Int.	.83	.89	.63	.47	.88	–	.64	.46	.67	.52	.71	.61	.68	.51	.68	.63	.40	.34	.45
7 – STARS‐M – Test/class	.71	.57	.80	.42	.90	.67	–	.51	.84	.85	.69	.58	.65	.68	.58	.41	.44	.31	.42
8 – STARS‐M – Help	.66	.48	.49	.86	.75	.53	.55	–	.51	.42	.52	.43	.41	.35	.46	.28	.47	.35	.41
9 – R‐MARS	.72	.63	.72	.43	.85	.72	.85	.55	–	.91	.87	.84	.72	.68	.70	.55	.43	.34	.45
10 – R‐MARS – Test	.62	.48	.74	.35	.77	.55	.86	.48	.92	–	.65	.60	.65	.73	.53	.40	.41	.28	.37
11 – R‐MARS – Course	.71	.67	.61	.51	.81	.76	.71	.58	.87	.67	–	.73	.68	.52	.79	.54	.39	.37	.46
12 – R‐MARS – Num.	.55	.59	.44	.30	.64	.67	.54	.38	.80	.56	.71	–	.58	.45	.58	.57	.31	.26	.36
13 – R‐MARS‐S	.86	.77	.86	.52	.78	.73	.74	.50	.77	.72	.69	.57	–	.89	.87	.84	.41	.30	.47
14 – R‐MARS‐S – Test	.78	.62	.89	.44	.71	.57	.76	.44	.73	.78	.55	.45	.92	–	.66	.59	.42	.26	.41
15 – R‐MARS‐S – Course	.82	.78	.72	.57	.74	.75	.62	.53	.69	.54	.78	.56	.86	.68	–	.66	.39	.31	.42
16 – R‐MARS‐S Num.	.65	.68	.54	.38	.60	.66	.47	.37	.58	.44	.57	.59	.79	.54	.66	–	.26	.23	.41
17 – Trait anxiety	.50	.39	.46	.48	.49	.38	.44	.46	.41	.38	.40	.26	.43	.42	.43	.25	–	.69	.72
18 – State anxiety (pre‐test)	.43	.36	.37	.40	.42	.36	.35	.39	.34	.29	.36	.26	.36	.33	.40	.24	.76	–	.74
19 – State anxiety (in‐test)	.50	.41	.44	.44	.50	.42	.44	.44	.44	.37	.44	.35	.46	.41	.48	.33	.70	.79	–

*Note*: All correlations were statistically significant at the *p* < .001 level.

Abbreviations: Help, Fear of asking for help; Int., Interpretation anxiety; Num., Numerical task anxiety; Test/class, Test and class anxiety.

### Original STARS and R‐MARS factor structures

We first checked how well the original STARS and R‐MARS factor structures fit the Study 1 data using Confirmatory Factor Analysis (CFA). CFA was conducted using the lavaan package (Rosseel, 2012), with polychoric correlations and the Weighted Least Squares with Mean and Variance adjustment (WLSMV) estimator to account for the ordinal Likert‐scale data. Measurement models were specified with standardized latent factors.

Both scales showed acceptable fit, with scaled CFI and TLI values at .95, SRMR values <.08 and RMSEA values within the .08–.10 range (Table 4). The *χ* ^2^ tests were statistically significant for both scales (Table 4), which is expected in large samples (*N* = 489; Kenny, 2020; Tanaka, 1987). Establishing this baseline fit demonstrated both instruments functioned as previously demonstrated in our context, meaning subsequent evidence of overlap cannot be attributed to the measures failing in our sample.

**TABLE 4:** Global fit statistics for CFAs of the original STARS and R‐MARS.

Scale	*χ* ^2^ (*df*, *N* = 489)	CFI	TLI	RMSEA [90% CI]	SRMR
STARS	1010.23 (227), *p* < .001	.95	.95	.08 [.08, .09]	.06
R‐MARS	916.29 (167), *p* < .001	.95	.95	.10 [.09, .10]	.06

Abbreviations: 90% CI, 90% confidence interval; CFI, comparative fit index; *df*, degrees of freedom; *p*, *p*‐value; RMSEA, root mean squared error of approximation; SRMR, standardized root mean squared residual; TLI, Tucker–Lewis index; *χ* ^2^, Chi‐squared difference test.

### Exploratory factor analysis

We next conducted exploratory factor analyses (EFA), first with only the original STARS and R‐MARS items. Most items clustered by their original subscales (Test and Class Anxiety, Interpretation Anxiety and Fear of Asking for Help from the STARS; and Mathematics Test Anxiety, Numerical Task Anxiety and Mathematics Course Anxiety from the R‐MARS), except for a Course Anxiety factor mixing both (Appendix S1, Section D). To determine whether this clustering reflected genuine domain specificity (statistics vs. mathematics), as opposed to similarities in item content, we repeated the EFA including the parallel versions of the STARS and R‐MARS. We did so following the logic that domain‐specific anxieties would produce separate factors, whereas task‐driven anxieties would yield factors comprising both statistics‐ and mathematics‐labelled items.

Before EFA, we assessed multicollinearity, which was expected given overlap between STARS and R‐MARS item content. The determinant was <.00001 and Bartlett's test was significant, indicating potential multicollinearity. Item‐level polychoric correlations (Appendix S1, Section C) showed 1.2% extremely high (*r*_pc_ > .90) and 4.5% high correlations (*r*_pc_ > .70), alongside 1.3% low correlations (*r*_pc_ < .20), indicating sufficient inter‐item association for factor extraction. The overall KMO indicated excellent sampling adequacy (MSA = .97), with comparable item‐level values, supporting estimation of distinct and reliable factors.

Analyses used the psych package (Revelle, 2019), polychoric correlations and minres extraction, consistent with ordinal data. The number of factors was guided by parallel analysis (PA), Very Simple Structure (VSS), Minimum Average Partial (MAP), RMSEA, SRMR, BIC and SABIC. These criteria disagreed, with PA suggesting 15 factors, VSS one or two and the others eight. Although VSS suggested one or two factors, the high item complexity in these solutions indicated that such broad models would collapse distinct anxiety experiences and were therefore not psychometrically interpretable. The 8‐ and 15‐factor solutions produced broadly similar clustering. The only substantive difference was that test‐anxiety items were split across two factors in the eight‐factor solution and fragmented into seven narrower clusters in the 15‐factor solution, none with clear theoretical distinctions. As this overfactoring likely reflected noise rather than distinct constructs, we retained the eight‐factor solution.

The eight‐factor solution was rotated obliquely (oblimin), with communalities based on squared multiple correlations. Items loadings of |λ| ≥ .40 were interpreted as salient. The resulting factors largely aligned with the substantive shared educational tasks and experiences reflected in the existing subscales from the original measures, but crucially did so across domains rather than separately for statistics and mathematics. As shown in Table 5, factors reflected construct‐level themes common to both scales, including Anticipatory Test Anxiety, Numeric Interpretation Anxiety, Fear of Asking for Help (particularly from teachers), Test Result Anxiety and Course Participation Anxiety. In each case, items originating from the STARS and R‐MARS loaded together on the same factors, indicating shared underlying anxieties rather than domain‐specific clustering.

**TABLE 5:** Results of the Study 1 exploratory factor analysis 8‐factor solution.

Item	1	2	3	4	5	6	7	8
Factor 1: Anticipatory test anxiety								
STARS – Test: Waking up in the morning on the day of a statistics test	.**80**	.13	.04	−.06	−.03	.08	.05	.03
R‐MARS‐S – Test: Thinking about an upcoming statistics test 1 h before	.**80**	.02	.08	−.03	.06	.06	−.03	.05
R‐MARS‐S – Test: Thinking about an upcoming statistics test 1 day before	.**75**	.08	.08	.11	.00	.00	−.02	.10
STARS – Test: Walking into the room to take a statistics test	.**67**	.10	.06	−.02	.02	.14	−.08	.03
STARS – Test: Doing an examination in a statistics course	.**65**	.14	.12	−.04	.13	.12	−.06	−.03
R‐MARS – Test: Thinking about an upcoming maths test 1 day before	.**65**	−.03	.05	.25	−.10	.01	.25	−.09
R‐MARS‐S – Test: Taking an exam (final) in a statistics course	.**65**	−.05	.05	−.05	.28	.20	−.16	−.08
R‐MARS‐S – Test: Thinking about an upcoming statistics test 1 week before	.**62**	.05	.06	.19	.07	−.07	.06	.17
STARS‐M – Test: Waking up in the morning on the day of a maths test	.**57**	.11	.06	.14	−.14	.19	.13	−.19
R‐MARS‐S – Test: Taking an exam (quiz) in a statistics course	.**57**	.04	.04	.01	.17	.17	.03	.09
R‐MARS – Test: Thinking about an upcoming maths test 1 h before	.**55**	−.03	.04	.15	−.08	.20	.21	−.20
R‐MARS – Test: Thinking about an upcoming maths test 1 week before	.**53**	.06	.06	.31	−.15	−.04	.25	−.05
R‐MARS‐S – Test: Taking the statistics section of a university entrance exam	.**53**	.01	.00	.03	.25	.14	.05	−.02
R‐MARS – Test: Taking an exam (final) in a maths course	.**52**	−.07	.03	.14	.04	.19	.14	−.35
STARS‐M – Test: Walking into the room to take a maths test	.**47**	.09	.11	.09	−.13	.23	.15	−.20
STARS – Test: Studying for an examination in a statistics course	.**46**	.14	.03	.32	.11	.03	−.15	.12
R‐MARS‐S – Test: Being give a surprise test in a statistics class	.**45**	.00	.02	.02	.18	.31	.04	.07
STARS‐M – Test: Doing an examination in a maths course	.**44**	.07	.02	.18	−.01	.24	.15	−.29
R‐MARS‐S – Test: Studying for a statistics test	.**42**	.16	.03	.32	.15	.06	−.12	.18
R‐MARS – Test: Taking an exam (quiz) in a maths course	.**42**	−.08	.03	.15	.09	.21	.34	−.19
Factor 2: Numerical interpretation anxiety								
STARS‐M – Int: Interpreting numbers in a table in a journal article	−.05	.**77**	.01	.12	.04	.06	.07	−.07
STARS‐M – Int: Trying to understand numerical information described in an article	−.02	.**73**	.05	.14	.10	.01	.05	−.15
STARS – Int: Interpreting the meaning of a table in a journal article	.14	.**66**	.05	−.01	.14	−.06	−.07	.13
STARS‐M – Int: Reading a journal article that includes some mathematical analyses	−.08	.**66**	.03	.12	.08	.10	.06	.00
STARS – Int: Trying to understand the statistical analyses described in the abstract of a journal article	.18	.**65**	.04	.08	.05	−.01	−.01	.11
STARS‐M – Int: Making an objective decision based on numerical information	−.02	.**58**	.08	.08	.15	.05	.12	−.17
STARS – Int: Reading a journal article that includes some statistical analyses	.15	.**57**	.07	.03	.11	−.02	.03	.21
STARS – Int: Making an objective decision based on empirical data	.19	.**56**	.02	−.04	.22	−.04	.07	.07
STARS – Int: Interpreting the meaning of a probability value once I have found it	.16	.**55**	.06	−.14	.23	.01	.11	.07
STARS‐M – Int: Determining whether a mathematical statement is true or false	−.08	.**51**	.06	.11	.08	.28	.12	−.16
STARS – Int: Trying to decide which analysis is appropriate for my research project	.20	.**49**	.17	−.06	.15	.07	−.23	.04
STARS‐M – Int: Interpreting the probability of it raining on a weather app	−.06	.**48**	−.04	.00	−.02	.08	.**41**	.12
STARS – Int: Trying to understand the odds in a lottery	−.04	.**47**	.02	−.01	.07	.11	.16	.17
STARS – Int: Determining whether to reject or retain the null hypothesis	.14	.**44**	.11	−.06	.11	.02	.12	.07
Factor 3: Fear of asking for help								
STARS – Help: Going to ask my statistics teacher for individual help with material I am having difficulty understanding	.08	−.08	.**94**	−.01	.05	−.08	−.08	.02
STARS – Help: Asking one of my teachers for help in understanding statistical output	.05	−.06	.**93**	−.04	.02	−.05	−.03	.11
STARS‐M – Help: Going to ask my maths teacher for individual help with material I am having difficulty understanding	−.01	−.04	.**91**	.08	−.01	.01	−.01	−.16
STARS‐M – Help: Asking one of my teachers for help in understanding a mathematical solution	−.04	−.04	.**90**	.04	−.02	.03	.09	−.12
STARS – Help: Asking someone in the computer lab for help in understanding statistical output	.03	.04	.**89**	−.07	−.02	.01	−.05	.13
STARS – Help: Asking a fellow student for help in understanding output	−.06	.07	.**81**	−.10	.06	.06	−.05	.13
STARS‐M – Help: Asking someone in the computer lab for help in understanding a mathematical solution	−.01	.06	.**81**	.05	−.05	.01	.03	−.07
STARS‐M – Help: Asking a fellow student for help in understanding a mathematical solution	−.11	.11	.**80**	−.01	−.04	.07	.08	−.09
Factor 4: Course anxiety								
STARS‐M – Test: Enrolling in a maths course	−.06	.07	.07	.**74**	.03	.10	.07	.01
R‐MARS – Course: Signing up for a maths course	−.01	.05	.10	.**68**	.08	.03	.14	−.01
R‐MARS‐S – Course: Signing up for a statistics course	.10	.11	.04	.**59**	.22	−.01	−.10	.20
R‐MARS – Test: Realizing you have to take a certain number of maths classes to fulfil requirements for your degree	.06	.02	.04	.**58**	.27	.04	.09	−.11
STARS – Test: Enrolling in a statistics course	.19	.14	.04	.**54**	.14	.02	−.10	.19
STARS‐M – Test: Doing the coursework for a maths course	.14	.26	.06	.**50**	.04	.11	−.09	−.20
STARS‐M – Test: Studying for an examination in a maths course	.24	.14	.05	.**47**	−.02	.12	.07	−.15
R‐MARS – Test: Studying for a maths test	.32	.05	.06	.**46**	.03	.05	.17	−.08
Factor 5: Statistical calculations anxiety								
R‐MARS‐S – Num: Calculating the variance of scores by dividing the sum of squared deviances by the number of scores	−.03	.04	.03	.01	.**83**	.04	.06	.02
R‐MARS‐S – Num: Calculating the sum of squared deviances by adding the squared deviances together	−.03	.03	.02	.00	.**83**	−.02	.14	.01
R‐MARS‐S – Num: Calculating the squared deviances by multiplying each deviance by itself	−.09	.08	.02	.07	.**81**	.07	.07	−.04
R‐MARS‐S – Num: Calculating the deviances of a set of scores on paper, with each deviance being the difference between the mean of the scores and each individual score in the set	.00	.09	.06	.07	.**77**	−.02	.01	−.05
Factor 6: Test result anxiety								
STARS‐M – Test: Going over a final examination in maths after it has been marked	.05	−.02	.08	.05	−.01	.**64**	.13	.08
STARS‐M – Test: Finding that another student in class got a different answer than I did to a mathematical problem	−.07	.25	.11	.09	−.02	.**64**	−.09	−.08
STARS – Test: Going over a final examination in statistics after it has been marked	.14	.01	.09	−.06	.11	.**59**	−.01	.24
R‐MARS – Test: Receiving your final maths grade	.23	−.15	.09	.04	.04	.**55**	.12	−.19
STARS – Test: Finding that another student in class got a different answer than I did to a statistical problem	.10	.19	.16	−.06	.07	.**53**	−.15	.08
Factor 7: Mathematical calculation anxiety								
R‐MARS – Num: Being given a set of subtraction problems to solve	.03	.08	.03	.05	.20	.04	.**69**	−.01
R‐MARS – Num: Being given a set of multiplication problems to solve	.11	.11	−.02	.03	.27	.02	.**64**	−.04
R‐MARS – Num: Being given a set of numerical problems involving addition to solve on paper	.08	.07	.01	.10	.25	.04	.**59**	.00
R‐MARS – Num: Being given a set of division problems to solve	.19	.21	.00	.01	.22	−.07	.**57**	−.08
R‐MARS – Num: Reading a cash register receipt after your purchase	−.08	.25	.17	−.10	.06	−.01	.**51**	.21
R‐MARS – Course: Buying a maths textbook	−.09	−.01	.20	.34	−.04	.08	.**41**	.37
Factor 8: N/A								
R‐MARS‐S – Course: Buying a statistics textbook	−.05	.07	.10	.31	.04	.07	.25	.**46**
Stray items								
STARS – Int: Watching a student search through a load of computer output from his/her research	.07	.39	.05	.13	−.03	.24	.06	.37
R‐MARS‐S – Course: Walking into a statistics class	.21	−.08	.24	.19	.23	.09	.14	.31
R‐MARS‐S – Course: Watching a teacher work on a statistical equation on the board	.05	.22	.10	.23	.26	.15	−.03	.28
STARS – Int: Reading an advertisement for a car which includes figures on miles per gallon, depreciation, etc.	.05	.39	.10	−.08	−.07	.06	.34	.25
STARS‐M – Int: Watching a student search through a load of computer output from his/her maths project	−.06	.37	.06	.22	−.07	.28	.13	.22
R‐MARS‐S – Course: Listening to another student explain a statistics formula	.10	.26	.22	.09	.24	.11	−.03	.22
STARS – Int: Arranging to have a body of data put into the computer	.12	.36	.19	.15	.14	.01	.02	.20
R‐MARS‐S – Test: Realizing you have to take a certain number of statistics classes to fulfil requirements for your degree	.22	.08	.01	.37	.37	.04	−.07	.16
R‐MARS – Course: Walking into a maths class	.03	−.05	.29	.32	.10	.10	.32	.08
R‐MARS – Course: Watching a teacher work on an algebraic equation on the board	−.09	.15	.09	.29	.11	.26	.30	.07
STARS – Test: Doing the coursework for a statistics course	.35	.23	.13	.35	.11	−.03	−.24	.05
R‐MARS – Course: Listening to another student explain a maths formula	−.05	.22	.22	.30	.06	.12	.21	.03
STARS‐M – Int: Trying to decide how to approach a mathematical problem to solve it	−.08	.37	.15	.29	.06	.23	.13	−.10
R‐MARS – Test: Being given a surprise test in a maths class	.31	−.05	.06	.20	.02	.27	.22	−.21
R‐MARS – Test: Taking the maths section of a university entrance exam	.35	−.03	.08	.16	.09	.19	.19	−.24

*Note*: Bold = factor loadings > |.40|.

Abbreviations: R‐MARS/R‐MARS‐S Course, R‐MARS/R‐MARS‐S Course Anxiety subscale item; R‐MARS/R‐MARS‐S Num, R‐MARS/R‐MARS‐S Numerical Task Anxiety subscale item; R‐MARS/R‐MARS‐S Test, R‐MARS/R‐MARS‐S Mathematics Test Anxiety subscale item; STARS/STARS‐M Help, STARS/STARS‐M Fear of Asking for Help subscale item; STARS/STARS‐M Int, STARS/STARS‐M Interpretation Anxiety subscale item; STARS/STARS‐M Test, STARS/STARS‐M Test and Class Anxiety subscale item.

The only clear domain separation emerged for Numerical Task Anxiety items from the R‐MARS and R‐MARS‐S, which split into Mathematical Calculation Anxiety and Statistical Calculation Anxiety. An eighth factor consisted of a single item and was therefore deemed spurious, yielding seven interpretable substantive factors. Several additional items also failed to load saliently onto any interpretable factor and were therefore not retained within the final substantive factor interpretation (see Table 5, ‘Stray Items’).

Overall, this pattern provides further evidence that statistics and mathematics anxiety overlap substantially at the level of several educational experiences, with limited domain specificity.

### Confirmatory factor analysis of the EFA structure

We re‐estimated the seven‐factor measurement model from Study 1 in the Study 2 sample (*n* = 245; polychoric/WLSMV). A warning indicated that the estimated latent covariance matrix was non–positive‐definite. However, there were no Heywood cases, and all latent factor correlations were <.80. The model was highly parameterized (≈325 free parameters) relative to the sample, which likely contributed to numerical fragility. Importantly, the smallest eigenvalue of the latent covariance matrix was −2.4 × 10^−17^ (≈0), indicating a numerical artefact rather than a substantive misspecification. Accordingly, we retained the seven‐factor model, but interpret the Study 2 estimates with appropriate caution.

Global fit was acceptable on robust (scaled) indices: *χ* ^2^(1994) = 3630.56, *p* < .001; CFI = .93; TLI = .93; RMSEA = .06, 90% CI [.05, .06]; SRMR = .08. The significant *χ* ^2^ is expected with large *df*; CFI/TLI ≈ .93 and RMSEA = .06 (with a narrow CI) indicate adequate fit, with SRMR at the conventional upper bound. For local fit, all items loaded strongly (|λ| ≥ .59) on their designated factors. Taken together, the seven‐factor structure from Study 1 generalized to the Study 2 sample.

### Latent profile analysis

Latent profile analyses (LPA) were conducted on mean‐centred composite scores for the STARS, R‐MARS and their modified forms (STARS‐M, R‐MARS‐S) using the tidyLPA package (Rosenberg et al., 2018). No missing data were present. Models with 1–6 profiles were estimated and compared using AIC, BIC, SABIC, entropy, posterior probabilities and the BLRT. SABIC was prioritized in line with recommendations from Ferguson et al. (2019).

In Study 1, the six‐profile solution was preferred because it showed the lowest information criteria values overall while retaining acceptable entropy (>.80) and posterior probabilities (>.70), indicating low profile uncertainty (Appendix S1, Section E, Table E1). Although one profile (Profile 5) represented <5% of the sample, we retained this solution because the profile remained evident in the *k* + 1 solution, supporting its stability rather than suggesting a spurious extraction (Ferguson et al., 2019). Importantly, this profile showed elevated mathematics anxiety alongside comparatively low statistics anxiety, representing one of the few patterns suggestive of dissociation between the constructs. As identifying potential asymmetries between mathematics anxiety and statistics anxiety was central to the aims of the present study, retaining a solution capable of capturing such differentiation was considered preferable to adopting more parsimonious solutions in which these distinctions were obscured in favour of broader and largely parallel anxiety profiles. Variance–covariance structures were compared (Masyn, 2013; Appendix S1, Section E, Table E2), and Model 2 (varying variances, zero covariances) was selected as optimal.

The same procedure in Study 2 again supported a six‐profile solution (Appendix S1, Section E, Tables E3 and E4). Profile patterns were also broadly mirrored across Studies 1 and 2 (Figures 1 and 2; Appendix S1, Section E, Tables E5 and E6), each with only one small profile showing higher mathematics than statistics anxiety. No profile in either sample indicated the reverse pattern.

**FIGURE 1:**
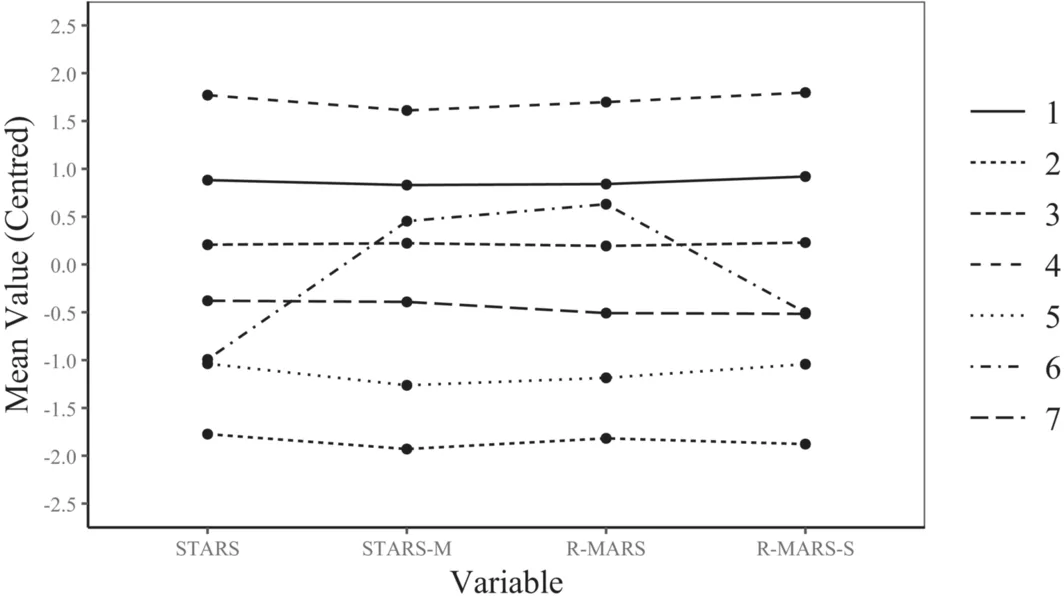
Means (centred) plot for the six‐profile solution in the Study 1 sample. Lines represent latent profiles. Scores are centred around the sample mean.

**FIGURE 2:**
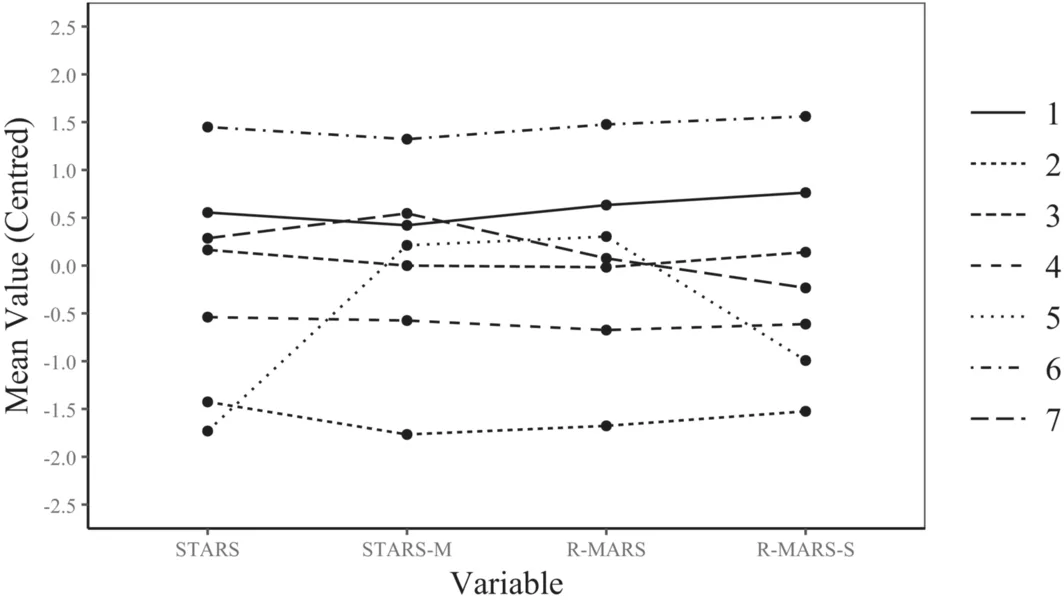
Means (centred) plot for the six‐profile solution in the Study 2 sample. Lines represent latent profiles. Scores are centred around the sample mean.

Overall, across both universities, ~95% of participants reported statistics and mathematics anxiety at comparable levels, suggesting it is rare for an individual to experience high statistics anxiety in the absence of mathematics anxiety.

### Multilevel modelling of experimental data

To test domain‐specific predictive validity, we fitted two three‐way multilevel models to the experimental data from each study. State anxiety (pre‐test/in‐test) was the outcome and MCQ condition (statistics vs. mathematics) was the between‐person factor. Model 1 included statistics anxiety (STARS) and its interactions with time and condition. Model 2 included mathematics anxiety (R‐MARS) and its interactions with time and condition. General trait anxiety was included as a covariate. Random intercepts and random slopes for time were specified (see Equations 1 and 2). Missingness was minimal (≤3.1% in Study 1; ≤1.2% in Study 2) so composite scores used available item means (Parent, 2013). STARS and R‐MARS were standardized, and trait anxiety was grand mean centred. Models were estimated with the lme4 (Bates et al., 2015) and lmerTest (Kuznetsova et al., 2017) packages. The assumptions of constant variance of the error term and normally distributed error terms were upheld in Models 1 and 2 in both Studies. See Appendix S1, Section F for descriptive statistics by condition for Study 1 (Table F1) and Study 2 (Table F2).

(1) $$\begin{array}{cc} \text{StateAn}x_{\mathrm{it}}= & \gamma_{00}+\gamma_{01}\left(\text{TraitAn}x_{i}\right)+\gamma_{02}\left(\text{MCQTyp}e_{i}\right)+\gamma_{03}\left(\text{StatsAn}x_{i}\right)\\ & +\gamma_{04}\left(\text{MCQTyp}e_{i}\times \text{StatsAn}x_{i}\right)+(\gamma_{10}+\gamma_{11}\left(\text{TraitAn}x_{i}\right)\\ & +\gamma_{12}\left(\text{MCQTyp}e_{i}\right)+\gamma_{13}\left(\text{StatsAn}x_{i}\right)\\ & +\gamma_{14}\left(\text{MCQTyp}e_{i}\times \text{StatsAn}x_{i}\right))\left(\mathrm{Tim}e_{\mathrm{it}}\right)+r_{0i}+r_{1i}\left(\mathrm{Tim}e_{\mathrm{it}}\right)+e_{\mathrm{it}} \end{array}$$

(2) $$\begin{array}{cc} \text{StateAn}x_{\mathrm{it}}= & \gamma_{00}+\gamma_{01}\left(\text{TraitAn}x_{i}\right)+\gamma_{02}\left(\text{MCQTyp}e_{i}\right)+\gamma_{03}\left(\text{MathsAn}x_{i}\right)\\ & +\gamma_{04}\left(\text{MCQTyp}e_{i}\times \text{MathsAn}x_{i}\right)\\ & +(\gamma_{10}+\gamma_{11}\left(\text{TraitAn}x_{i}\right)+\gamma_{12}\left(\text{MCQTyp}e_{i}\right)+\gamma_{13}\left(\text{MathsAn}x_{i}\right)\\ & +\gamma_{14}\left(\text{MCQTyp}e_{i}\times \text{MathsAn}x_{i}\right))\left(\mathrm{Tim}e_{\mathrm{it}}\right)+r_{0i}+r_{1i}\left(\mathrm{Tim}e_{\mathrm{it}}\right)+e_{\mathrm{it}} \end{array}$$

To determine the extent to which the tasks elicited state anxiety, we examined change in state anxiety from pre‐ to in‐test. In Study 1, changes in state anxiety were small and non‐statistically significant overall (βs = .04, *p*s ≥ .137). In contrast, Study 2 showed modest but statistically significant increases in state anxiety over time (βs = .10–.11, *p*s ≤ .013), indicating that the task elicited a general increase in anxiety across conditions. However, these increases did not differ according to congruence between domain‐specific anxiety and MCQ condition, as reflected in the small and non‐significant three‐way interaction terms (reported below).

Across the two samples, higher levels of statistics or mathematics anxiety were associated with larger pre‐ to in‐test increases in state anxiety, with comparable effect size estimates across Models 1 and 2 (time × STARS: β = .08–.14, *p*s ≤ .002; time × R‐MARS: β = .08–.13, *p*s ≤ .003; Appendix S1, Section F, Tables F3–F6).

By contrast, estimates for the three‐way interactions (time × anxiety × MCQ condition) were small and inconsistent across samples (|β| ≤ .05, *p*s ≥ .32), providing no evidence that anxiety‐related changes in state anxiety differed by MCQ domain (Appendix S1, Section F, Figures F1–F4). In other words, individuals high in statistics anxiety became more anxious during testing regardless of whether they were exposed to the statistics or mathematics MCQs and likewise for mathematics anxiety.

## DISCUSSION

Our investigation into the distinctiveness of the STARS and R‐MARS provides converging evidence of a jangle fallacy (when two measures with different names capture the same underlying construct; Kelley, 1927). Across multiple methods—correlations, factor analyses, latent profiles and an experimental test of predictive validity—we found little evidence that the two scales capture genuinely independent constructs.

### Strong structural associations

The composite‐level correlations between the STARS and R‐MARS were notably strong (*r* = .68 to .85 across both samples), similar to those between the mathematics and statistics scales reported by Tremblay et al. (2000; *r* = .81), Gibeau et al. (2023; *r* = .79) and Primi & Chiesi (2018; *r* = .61).

Lower correlations reported elsewhere (e.g., Birenbaum & Eylath, 1994; Zeidner, 1991) may reflect methodological artefacts. For example, Zeidner (1991) and Birenbaum and Eylath (1994) compared multi‐item scales to single‐item measures, conflating construct and method variance. By creating parallel, content‐matched versions of the STARS and R‐MARS—in which item wording was adapted to refer to the alternate domain—we showed that subscales measuring the same type of anxiety (e.g., test‐, course‐ or numerically focused anxiety) were consistently and strongly correlated across domains (typically *r* ≈ .66–.92), with substantially less unique variance remaining once content mismatches were removed. This pattern indicates that much of the apparent distinctiveness between statistics and mathematics anxiety reflects differences in item content rather than construct‐specific variance. Consistent with benchmarks proposed by Lawson and Robins (2021), whereby correlations of |.80| and above indicate substantial overlap, these findings suggest that the distinctiveness reported in earlier studies was largely methodological in origin.

### Shared factor structure

Our exploratory factor analyses further undermined the claim of distinctiveness. Five of the seven interpretable factors contained items from both the STARS and R‐MARS, indicating that much of the shared variance reflects anxieties associated with shared educational tasks and activities common to both domains, rather than discipline‐specific concerns. These included factors related to classroom participation (e.g., asking for help), assessment contexts (e.g., test anticipation) and engagement with quantitative information, all of which occur in both mathematics and statistics learning contexts. This pattern is more consistent with a task‐driven account of educational anxiety than a domain‐driven account, because anxieties clustered around shared learning practices irrespective of disciplinary label, rather than forming distinct, domain‐specific factors. This contrasts with Paechter et al. (2017), who could not reveal cross‐loading patterns because the scales were examined separately.

The only two factors that separated clearly were Mathematical Calculation Anxiety and Statistical Calculation Anxiety. These results may indicate some degree of differentiation between statistics and mathematics anxieties in relation to calculation tasks, which is broadly consistent with suggestions that different types of calculation can engage different cognitive processes and therefore elicit domain‐specific anxieties (Baloğlu, 2004; Ben‐Zvi & Garfield, 2008; Hunt et al., 2019). However, we caution that the items we reworded for the modified versions of the STARS and R‐MARS may have introduced differences in familiarity, technicality or difficulty (e.g., ‘Being given a set of multiplication problems to solve’ versus ‘Calculating the squared deviances by multiplying each deviance by itself’), which could explain some separation. Consequently, although the calculation factors may suggest some level of domain specificity, they should not be interpreted as definitive evidence that statistics and mathematics anxieties are distinct constructs.

### Co‐occurrence of anxieties

Latent profile analyses provided evidence that statistics and mathematics anxieties tend to exist in tandem. In both samples, more than 94% of students showed parallel levels of anxiety across scales. Profiles of high mathematics but low statistics anxiety were rare, and no profiles emerged with the reverse pattern (high statistics but low mathematics anxiety). Overall, these findings suggest that statistics anxiety seldom arises in the absence of mathematics anxiety and is therefore more plausibly interpreted as a manifestation of it. The small number of exceptions may reflect differences in educational context or prior experience. For example, students with more advanced mathematics training may be better able to distinguish statistics from mathematics conceptually, resulting in somewhat different emotional responses to each domain. Future qualitative work could explore these exceptions to identify explanatory factors (e.g., curriculum differences, prior mastery), followed by quantitative tests of their predictive value across education systems.

### Lack of predictive specificity

Our experimental test of predictive validity also failed to support distinctiveness. Higher levels of statistics or mathematics anxiety predicted larger increases in state anxiety during the MCQ task. However, the magnitude of these increases did not depend on whether participants completed a mathematics or statistics version of the MCQ. This pattern mirrors the latent profile analysis results: because most participants showed aligned levels of statistics and mathematics anxiety, their state anxiety responses did not diverge across test domains. While the MCQs were intended to provide discipline‐specific evaluative contexts in which state anxiety responses could be observed, the observed increases were generally modest and somewhat inconsistent across studies. Statistically significant overall increases were observed in Study 2 but not Study 1. This discrepancy may reflect sampling variation or differences in responsiveness to the experimental context (e.g., students at University B may have been more likely to experience anxiety in testing situations). Importantly, these differences do not undermine our conclusions regarding distinctiveness, as the critical interaction terms remained small and non‐significant across both studies.

One possible explanation for the modest increases is that anticipatory test anxiety elevated pre‐task state anxiety scores because participants knew they would later complete a multiple‐choice test. Research suggests that anxiety responses can vary across anticipatory, test‐taking and post‐test phases, with anxiety often emerging prior to task commencement as individuals anticipate and appraise an upcoming evaluative situation (Zeidner, 1998). If anxiety was already elevated at the pre‐task measurement point, that could leave relatively little scope for additional increases during test completion. However, participants were not informed whether they would complete the mathematics or statistics version of the task, making it unlikely that anticipatory anxiety introduced domain‐specific effects. Thus, although anticipatory anxiety may have attenuated overall changes in state anxiety, it is less likely to account for the absence of domain‐specific predictive effects.

We also cannot rule out the possibility that evaluative tasks with real‐world consequences might produce larger state anxiety responses than those observed here. Future research should test whether more ecologically valid stressors—such as graded assessments or time‐pressured problem‐solving—might reveal differences.

### Implications

Statistics anxiety is often treated as a construct distinct from mathematics anxiety, yet the present findings point to substantial overlap between statistics anxiety and mathematics anxiety. This overlap was evident across multiple educational experiences, including tests, classroom participation, interpretation and help‐seeking, suggesting that many anxieties commonly attributed to statistics are not unique to that domain. At the same time, there was tentative evidence of differentiation for anxieties associated with calculation tasks, although it remains unclear whether this reflects genuine domain specificity or differences in the nature of the calculation items themselves. Further, our findings indicated the existence of a small sub‐sample who experienced high statistics but low mathematics anxiety, but no cases of the opposite pattern. This dissociation presents a fascinating conundrum. Accordingly, statistics anxiety may be best conceptualized as a partially differentiated manifestation of broader mathematics‐related anxiety rather than a wholly separate construct. This interpretation has important implications for research, practice and policy.

For research, findings indicate treating statistics anxiety as distinct perpetuates a jangle fallacy that duplicates measures, fragments the literature and obscures theoretical progress. Including both scales in predictive models risks collinearity, while studies that claim to address statistics anxiety may in fact be targeting broader mathematics‐related difficulties. Recognizing the overlap can allow for more parsimonious measurement, clearer theory building and more accurate interpretation of findings.

For practice, our findings suggest that treating mathematics and statistics anxiety as separate targets is unlikely to add substantial value. That is, strategies intended to address mathematics anxiety are likely to generalize directly to statistics learning. This aligns with our psychometric findings, but may also reflect students' lived experiences: on many psychology courses, it is plausible that statistics is encountered primarily as another manifestation of mathematics, eliciting similar cognitive and affective responses.

Importantly, our results also indicate that what existing scales currently label as statistics or mathematics anxiety may reflect broader educational anxieties (e.g., related to classroom participation and assessment contexts). Inclusive and active teaching practices which aim to tackle more general academic anxieties may therefore act as a ‘Trojan horse’, simultaneously reducing anxiety surrounding mathematics and statistics. From this perspective, improving how statistics and mathematics are taught may be more impactful than attempting to treat students' anxieties as separate, subject‐specific problems.

For policy, the observed overlap indicates that efforts to strengthen numeracy confidence earlier in education could have downstream benefits for students' later engagement with statistics. If statistics anxiety is largely rooted in prior mathematics experiences, prevention may be more effective than attempting remediation once students reach higher education. At the institutional level, this also supports policies that promote inclusive and active learning approaches in quantitatively demanding university courses, aiming to reduce anxiety through course design rather than treating it solely as an individual deficit.

Taken together, these implications show that the question of construct distinctiveness is not merely academic. How we define and measure statistics anxiety shapes the theories we build, the supports, teaching and curricula we design and the policies we pursue. Situating statistics anxiety within the broader framework of mathematics anxiety—and, more broadly, within shared educational anxieties associated with learning and assessment—may therefore help reduce barriers to learning in numeracy‐rich domains.

### Limitations and future directions

Our samples consisted of psychology undergraduates from South‐East England, limiting generalizability. Students in different educational contexts—such as countries where mathematics is compulsory to age 18—may show different anxiety profiles. Larger, more diverse samples are therefore needed.

It could also be argued that our findings are specific to the STARS and R‐MARS. However, the only other statistics anxiety measure with accumulated validity evidence (the SAS; Vigil‐Colet et al., 2008) is derived directly from the STARS, and the R‐MARS subscales map closely onto other mathematics anxiety instruments (Luttenberger et al., 2018). We are, therefore, confident that our findings will generalize.

Descriptive analyses indicated lower performance on the statistics MCQs than the mathematics MCQs across both studies, particularly in Study 2 (see Appendix S1, Section A). However, despite these differences in task performance, the pattern of state anxiety findings remained broadly similar across domains so does not readily explain the absence of domain‐specific anxiety effects. Future work could employ more tightly equated quantitative tasks under controlled testing conditions, using pilot testing to assess equivalence in both objective performance and perceived difficulty, and to examine how subjective appraisals of task demands relate to anxiety responses.

Finally, while the COVID‐19 pandemic may have elevated baseline anxiety levels (Pashazadeh Kan et al., 2021; Pennino et al., 2022), such effects were unlikely to be systematic and so cannot explain the high overlap observed.

## CONCLUSION

Counter to the prevailing narrative, our findings show that statistics anxiety—as measured by the STARS—is not distinct from mathematics anxiety—as measured by the R‐MARS. Across two undergraduate samples and multiple analytic approaches, the two overlapped extensively, with apparent differences potentially explained by scale content rather than true domain specificity. While rare exceptions may exist, the evidence suggests that treating statistics anxiety as independent risks fragmenting theory and duplicating measures, as well as misinforming policy, interventions and teaching practice. A more productive path is to recognize statistics anxiety as part of the broader mathematics anxiety construct, while also acknowledging its embedding within wider educational anxieties related to learning, assessment and classroom participation. Adopting a more holistic perspective would allow educational psychology to build clearer theories, design more efficient pedagogies and supports and better address one of the most pervasive barriers to student learning in numeracy‐rich domains.

## AUTHOR CONTRIBUTIONS

**Jenny Terry:** Conceptualization; methodology; data curation; formal analysis; investigation; visualization; project administration; writing – original draft; writing – review and editing; resources. **Charlie Lea:** Investigation; writing – review and editing. **Andy P. Field:** Conceptualization; methodology; supervision; resources; writing – review and editing.

## CONFLICT OF INTEREST STATEMENT

The authors have no conflicts of interest to declare.

## ETHICS STATEMENT

This study was approved (ER/JLT26/5) by the Sciences & Technology Cross‐Schools Research Ethics Committee (C‐REC) at the University of Sussex and adhered to the British Psychological Society's Code of Human Research Ethics (British Psychological Society [BPS] Ethics Board, 2018).

## Supporting information


Appendix S1

## Data Availability

The data that support the findings of this study are openly available on the OSF at https://doi.org/10.17605/OSF.IO/8JK5V.

## Footnotes

^1^
Students who took the survey as part of their assessment exercise also completed an additional questionnaire about statistics self‐efficacy that they had co‐created in a statistics class after the in‐test state anxiety measures. These students received no specific instruction about emotional‐motivational processes within that class. The self‐efficacy questionnaire was not used in the present study.
