# Supplementary material for: Welcome to the jangle (fallacy): The case of statistics and mathematics anxieties
Source: Br J Educ Psychol. 2026 Aug 16;96(4):1802–27. doi: 10.1111/bjep.70119 (PMC13752620; doi:10.1111/bjep.70119)
Supplement: Supplementary file 1 [file BJEP-96-1802-s001.docx]

**Appendix**

Contents

[Section A: MCQ Task Scoring	3](#_Toc237338745)

[Table A1	3](#_Toc237338746)

[Table A2	3](#_Toc237338747)

[Section B: Item Level Descriptive Statistics	4](#_Toc237338748)

[Table B1	4](#_Toc237338749)

[Section C: Item Level Polychoric Correlations	13](#_Toc237338751)

[Section D: EFA of the STARS & R-MARS	14](#_Toc237338752)

[Table D1	14](#_Toc237338753)

[Table D2	14](#_Toc237338754)

[Section E: Latent Profile Analysis	18](#_Toc237338755)

[Table E1	18](#_Toc237338756)

[Table E2	18](#_Toc237338757)

[Table E3	19](#_Toc237338758)

[Table E4	20](#_Toc237338759)

[Table E5	20](#_Toc237338760)

[Table E6	21](#_Toc237338761)

[Section F: Multi-Level Models	22](#_Toc237338762)

[Table F1	22](#_Toc237338763)

[Table F2	23](#_Toc237338764)

[Table F3	24](#_Toc237338765)

[Table F4	25](#_Toc237338766)

[Figure F1	26](#_Toc237338767)

[Figure F2	27](#_Toc237338768)

[Table F5	28](#_Toc237338769)

[Table F6	29](#_Toc237338770)

[Figure F3	30](#_Toc237338771)

[Figure F4	31](#_Toc237338772)

# Section A: MCQ Task Scoring

Appendix F presents descriptive statistics for performance on the mathematics and statistics MCQs across both studies. Although the MCQs were designed to be broadly parallel in content and structure, they were administered as brief low-stakes anxiety-induction tasks rather than standardised assessments of quantitative ability. Consequently, score differences should not necessarily be interpreted solely as reflecting objective task difficulty, as performance may also have been influenced by factors such as engagement, guessing, or anxiety-related disruption typical of unsupervised online testing contexts.

## Table A1

*Descriptive Statistics for Mathematics and Statistics MCQ Performance Across Studies.*

| Study | Condition | n | Mean | SD | Min | Max | Cohen’s d |
| --- | --- | --- | --- | --- | --- | --- | --- |
| Study 1 | Mathematics | 236 | 3.23 | 1.22 | 0 | 5 | 0.50 |
| Study 1 | Statistics | 240 | 2.64 | 1.12 | 0 | 5 |  |
| Study 2 | Mathematics | 122 | 2.44 | 1.25 | 0 | 5 | 0.73 |
| Study 2 | Statistics | 120 | 1.62 | 0.98 | 0 | 4 |  |

*Note.* Cohen’s d values represent the standardised difference between mathematics and statistics MCQ scores within each study. Higher scores indicate greater task accuracy.

## Table A2

*Percentage Correct for each Mathematics and Statistics MCQ Item Across Studies.*

| Question | Study 1 Statistics | Study 1 Mathematics | Study 2 Statistics | Study 2 Mathematics |
| --- | --- | --- | --- | --- |
| 1 | 83.5 | 73.9 | 67.5 | 63.1 |
| 2 | 38.1 | 80.8 | 45.0 | 67.5 |
| 3 | 32.3 | 51.3 | 22.1 | 42.5 |
| 4 | 48.9 | 49.8 | 5.9 | 40.6 |
| 5 | 59.5 | 64.1 | 22.0 | 47.5 |

# Section B: Item Level Descriptive Statistics

## **Table B1**

Descriptive Statistics for all Items Included in the EFAs.

| Item | Mean | *SD* | Min | Max | Skew | Kurtosis | *SE* |
| --- | --- | --- | --- | --- | --- | --- | --- |
| STARS - Test: Studying for an examination in a statistics course | 3.38 | 1.13 | 1.00 | 5.00 | -0.26 | -0.78 | 0.05 |
| STARS - Int: Interpreting the meaning of a table in a journal article | 2.70 | 1.16 | 1.00 | 5.00 | 0.24 | -0.85 | 0.05 |
| STARS - Help: Going to ask my statistics teacher for individual help with material I am having difficulty understanding | 3.04 | 1.32 | 1.00 | 5.00 | 0.02 | -1.17 | 0.06 |
| STARS - Test: Doing the coursework for a statistics course | 3.41 | 1.12 | 1.00 | 5.00 | -0.16 | -0.91 | 0.05 |
| STARS - Int: Making an objective decision based on empirical data | 2.73 | 1.18 | 1.00 | 5.00 | 0.13 | -0.94 | 0.05 |
| STARS - Int: Reading a journal article that includes some statistical analyses | 2.40 | 1.10 | 1.00 | 5.00 | 0.44 | -0.63 | 0.05 |
| STARS - Int: Trying to decide which analysis is appropriate for my research project | 3.52 | 1.06 | 1.00 | 5.00 | -0.31 | -0.56 | 0.05 |
| STARS - Test: Doing an examination in a statistics course | 3.95 | 1.05 | 1.00 | 5.00 | -0.75 | -0.31 | 0.05 |
| STARS - Int: Reading an advertisement for a car which includes figures on miles per gallon, depreciation, etc. | 1.99 | 1.11 | 1.00 | 5.00 | 0.92 | -0.04 | 0.05 |
| STARS - Test: Walking into the room to take a statistics test | 3.69 | 1.15 | 1.00 | 5.00 | -0.58 | -0.56 | 0.05 |
| STARS - Int: Interpreting the meaning of a probability value once I have found it | 2.61 | 1.20 | 1.00 | 5.00 | 0.28 | -0.92 | 0.05 |
| STARS - Int: Arranging to have a body of data put into the computer | 2.81 | 1.23 | 1.00 | 5.00 | 0.19 | -0.93 | 0.06 |
| STARS - Test: Finding that another student in class got a different answer than I did to a statistical problem | 3.39 | 1.08 | 1.00 | 5.00 | -0.18 | -0.75 | 0.05 |
| STARS - Int: Determining whether to reject or retain the null hypothesis | 2.59 | 1.18 | 1.00 | 5.00 | 0.33 | -0.79 | 0.05 |
| STARS - Test: Waking up in the morning on the day of a statistics test | 3.77 | 1.13 | 1.00 | 5.00 | -0.64 | -0.51 | 0.05 |
| STARS - Help: Asking one of my teachers for help in understanding statistical output | 2.83 | 1.24 | 1.00 | 5.00 | 0.15 | -0.99 | 0.06 |
| STARS - Int: Trying to understand the odds in a lottery | 2.12 | 1.14 | 1.00 | 5.00 | 0.79 | -0.28 | 0.05 |
| STARS - Int: Watching a student search through a load of computer output from his/her research | 2.54 | 1.28 | 1.00 | 5.00 | 0.37 | -1.01 | 0.06 |
| STARS - Help: Asking someone in the computer lab for help in understanding statistical output | 2.82 | 1.24 | 1.00 | 5.00 | 0.20 | -1.05 | 0.06 |
| STARS - Int: Trying to understand the statistical analyses described in the abstract of a journal article | 2.82 | 1.20 | 1.00 | 5.00 | 0.17 | -0.97 | 0.05 |
| STARS - Test: Enrolling in a statistics course | 2.76 | 1.28 | 1.00 | 5.00 | 0.21 | -1.02 | 0.06 |
| STARS - Test: Going over a final examination in statistics after it has been marked | 3.06 | 1.24 | 1.00 | 5.00 | 0.01 | -1.00 | 0.06 |
| STARS - Help: Asking a fellow student for help in understanding output | 2.64 | 1.21 | 1.00 | 5.00 | 0.33 | -0.90 | 0.05 |
| STARS-M - Test: Studying for an examination in a maths course | 3.22 | 1.17 | 1.00 | 5.00 | -0.10 | -0.90 | 0.05 |
| STARS-M - Int: Interpreting numbers in a table in a journal article | 2.57 | 1.14 | 1.00 | 5.00 | 0.29 | -0.81 | 0.05 |
| STARS-M - Help: Going to ask my maths teacher for individual help with material I am having difficulty understanding | 2.92 | 1.29 | 1.00 | 5.00 | 0.05 | -1.10 | 0.06 |
| STARS-M - Test: Doing the coursework for a maths course | 3.13 | 1.17 | 1.00 | 5.00 | -0.01 | -0.96 | 0.05 |
| STARS-M - Int: Making an objective decision based on numerical information | 2.64 | 1.11 | 1.00 | 5.00 | 0.21 | -0.74 | 0.05 |
| STARS-M - Int: Reading a journal article that includes some mathematical analyses | 2.45 | 1.12 | 1.00 | 5.00 | 0.44 | -0.61 | 0.05 |
| STARS-M - Int: Trying to decide how to approach a mathematical problem to solve it | 2.79 | 1.12 | 1.00 | 5.00 | 0.27 | -0.66 | 0.05 |
| STARS-M - Test: Doing an examination in a maths course | 3.74 | 1.16 | 1.00 | 5.00 | -0.54 | -0.79 | 0.05 |
| STARS-M - Test: Walking into the room to take a maths test | 3.61 | 1.19 | 1.00 | 5.00 | -0.50 | -0.72 | 0.05 |
| STARS-M - Int: Interpreting the probability of it raining on a weather app | 1.88 | 1.08 | 1.00 | 5.00 | 1.11 | 0.35 | 0.05 |
| STARS-M - Test: Finding that another student in class got a different answer than I did to a mathematical problem | 3.35 | 1.09 | 1.00 | 5.00 | -0.17 | -0.71 | 0.05 |
| STARS-M - Int: Determining whether a mathematical statement is true or false | 2.71 | 1.12 | 1.00 | 5.00 | 0.22 | -0.76 | 0.05 |
| STARS-M - Test: Waking up in the morning on the day of a maths test | 3.66 | 1.11 | 1.00 | 5.00 | -0.48 | -0.65 | 0.05 |
| STARS-M - Help: Asking one of my teachers for help in understanding a mathematical solution | 2.76 | 1.22 | 1.00 | 5.00 | 0.26 | -0.86 | 0.05 |
| STARS-M - Int: Watching a student search through a load of computer output from his/her maths project | 2.50 | 1.27 | 1.00 | 5.00 | 0.40 | -0.97 | 0.06 |
| STARS-M - Help: Asking someone in the computer lab for help in understanding a mathematical solution | 2.84 | 1.20 | 1.00 | 5.00 | 0.06 | -0.97 | 0.05 |
| STARS-M - Int: Trying to understand numerical information described in an article | 2.69 | 1.15 | 1.00 | 5.00 | 0.25 | -0.78 | 0.05 |
| STARS-M - Test: Enrolling in a maths course | 2.72 | 1.28 | 1.00 | 5.00 | 0.27 | -1.05 | 0.06 |
| STARS-M - Test: Going over a final examination in maths after it has been marked | 2.92 | 1.24 | 1.00 | 5.00 | 0.08 | -0.97 | 0.06 |
| STARS-M - Help: Asking a fellow student for help in understanding a mathematical solution | 2.52 | 1.15 | 1.00 | 5.00 | 0.42 | -0.67 | 0.05 |
| R-MARS - Test: Studying for a maths test | 2.94 | 1.09 | 1.00 | 5.00 | 0.09 | -0.69 | 0.05 |
| R-MARS - Test: Taking the maths section of a university entrance exam | 3.56 | 1.18 | 1.00 | 5.00 | -0.39 | -0.86 | 0.05 |
| R-MARS - Test: Taking an exam (quiz) in a maths course | 3.42 | 1.16 | 1.00 | 5.00 | -0.29 | -0.80 | 0.05 |
| R-MARS - Test: Taking an exam (final) in a maths course | 4.00 | 1.05 | 1.00 | 5.00 | -0.81 | -0.16 | 0.05 |
| R-MARS - Test: Thinking about an upcoming maths test 1 week before | 3.00 | 1.18 | 1.00 | 5.00 | 0.05 | -0.88 | 0.05 |
| R-MARS - Test: Thinking about an upcoming maths test 1 day before | 3.47 | 1.15 | 1.00 | 5.00 | -0.24 | -0.80 | 0.05 |
| R-MARS - Test: Thinking about an upcoming maths test 1 hour before | 3.76 | 1.10 | 1.00 | 5.00 | -0.53 | -0.59 | 0.05 |
| R-MARS - Test: Realizing you have to take a certain number of maths classes to fulfill requirements for your degree | 2.99 | 1.31 | 1.00 | 5.00 | 0.02 | -1.08 | 0.06 |
| R-MARS - Test: Receiving your final maths grade | 3.88 | 1.08 | 1.00 | 5.00 | -0.73 | -0.25 | 0.05 |
| R-MARS - Test: Being given a suprise test in a maths class | 3.81 | 1.11 | 1.00 | 5.00 | -0.60 | -0.56 | 0.05 |
| R-MARS - Num: Reading a cash register receipt after your purchase | 1.42 | 0.80 | 1.00 | 5.00 | 2.08 | 3.97 | 0.04 |
| R-MARS - Num: Being given a set of numerical problems involving addition to solve on paper | 2.05 | 1.14 | 1.00 | 5.00 | 0.94 | -0.04 | 0.05 |
| R-MARS - Num: Being given a set of subtraction problems to solve | 1.78 | 1.01 | 1.00 | 5.00 | 1.37 | 1.36 | 0.05 |
| R-MARS - Num: Being given a set of multiplication problems to solve | 2.09 | 1.14 | 1.00 | 5.00 | 0.88 | -0.06 | 0.05 |
| R-MARS - Num: Being given a set of division problems to solve | 2.29 | 1.14 | 1.00 | 5.00 | 0.60 | -0.46 | 0.05 |
| R-MARS - Course: Buying a maths textbook | 1.62 | 0.99 | 1.00 | 5.00 | 1.66 | 2.12 | 0.04 |
| R-MARS - Course: Watching a teacher work on an algebraic equation on the board | 2.37 | 1.14 | 1.00 | 5.00 | 0.54 | -0.57 | 0.05 |
| R-MARS - Course: Signing up for a maths course | 2.60 | 1.27 | 1.00 | 5.00 | 0.34 | -0.98 | 0.06 |
| R-MARS - Course: Listening to another student explain a maths formula | 2.45 | 1.18 | 1.00 | 5.00 | 0.45 | -0.71 | 0.05 |
| R-MARS - Course: Walking into a maths class | 2.22 | 1.16 | 1.00 | 5.00 | 0.78 | -0.27 | 0.05 |
| R-MARS-S - Test: Studying for a statistics test | 3.27 | 1.09 | 1.00 | 5.00 | -0.11 | -0.80 | 0.05 |
| R-MARS-S - Test: Taking the statistics section of a university entrance exam | 3.83 | 1.12 | 1.00 | 5.00 | -0.67 | -0.46 | 0.05 |
| R-MARS-S - Test: Taking an exam (quiz) in a statistics course | 3.60 | 1.12 | 1.00 | 5.00 | -0.38 | -0.77 | 0.05 |
| R-MARS-S - Test: Taking an exam (final) in a statistics course | 4.18 | 0.97 | 1.00 | 5.00 | -1.07 | 0.42 | 0.04 |
| R-MARS-S - Test: Thinking about an upcoming statistics test 1 week before | 3.19 | 1.16 | 1.00 | 5.00 | -0.12 | -0.80 | 0.05 |
| R-MARS-S - Test: Thinking about an upcoming statistics test 1 day before | 3.72 | 1.09 | 1.00 | 5.00 | -0.54 | -0.50 | 0.05 |
| R-MARS-S - Test: Thinking about an upcoming statistics test 1 hour before | 3.98 | 1.04 | 1.00 | 5.00 | -0.85 | -0.06 | 0.05 |
| R-MARS-S - Test: Realizing you have to take a certain number of statistics classes to fulfill requirements for your degree | 3.22 | 1.29 | 1.00 | 5.00 | -0.13 | -1.11 | 0.06 |
| R-MARS-S - Test: Receiving your final statistics grade | 4.06 | 1.03 | 1.00 | 5.00 | -0.93 | 0.13 | 0.05 |
| R-MARS-S - Test: Being give a surprise test in a statistics class | 4.12 | 1.02 | 1.00 | 5.00 | -0.96 | 0.09 | 0.05 |
| R-MARS-S - Num: Calculating the deviances of a set of scores on paper, with each deviance being the difference between the mean of the scores and each individual score in the set | 3.05 | 1.28 | 1.00 | 5.00 | -0.05 | -1.07 | 0.06 |
| R-MARS-S - Num: Calculating the squared deviances by multiplying each deviance by itself | 2.75 | 1.32 | 1.00 | 5.00 | 0.24 | -1.10 | 0.06 |
| R-MARS-S - Num: Calculating the sum of squared deviances by adding the squared deviances together | 2.70 | 1.31 | 1.00 | 5.00 | 0.28 | -1.06 | 0.06 |
| R-MARS-S - Num: Calculating the variance of scores by dividing the sum of squared deviances by the number of scores | 2.81 | 1.29 | 1.00 | 5.00 | 0.22 | -1.05 | 0.06 |
| R-MARS-S - Course: Buying a statistics textbook | 1.79 | 1.05 | 1.00 | 5.00 | 1.34 | 1.14 | 0.05 |
| R-MARS-S - Course: Watching a teacher work on a statistical equation on the board | 2.54 | 1.16 | 1.00 | 5.00 | 0.38 | -0.70 | 0.05 |
| R-MARS-S - Course: Signing up for a statistics course | 2.71 | 1.23 | 1.00 | 5.00 | 0.30 | -0.91 | 0.06 |
| R-MARS-S - Course: Listening to another student explain a statistics formula | 2.70 | 1.21 | 1.00 | 5.00 | 0.26 | -0.92 | 0.05 |
| R-MARS-S - Course: Walking into a statistics class | 2.38 | 1.18 | 1.00 | 5.00 | 0.64 | -0.42 | 0.05 |

*Note.* STARS/STARS-M Int = STARS/STARS-M Interpretation Anxiety subscale item; STARS/STARS-M Help = STARS/STARS-M Fear of Asking for Help subscale item; STARS/STARS-M Test = STARS/STARS-M Test and Class Anxiety subscale item; R-MARS/R-MARS-S Test = R-MARS/R-MARS-S Mathematics Test Anxiety subscale item; R-MARS/R-MARS-S Course = R-MARS/R-MARS-S Course Anxiety subscale item; R-MARS/R-MARS-S Num = R-MARS/R-MARS-S Numerical Task Anxiety subscale item; *SD* = standard deviation; Min = minimum recorded value; Max = maximum recorded value; *SE* = standard error.

# Section C: Item Level Polychoric Correlations

Table C1 shows the polychoric correlations between all items in the four statistics and mathematics anxiety scales included in the EFA. Due the size of Table C1, it is stored in a separate spreadsheet, available here: <https://osf.io/378zg/files/3uarz>.

# Section D: EFA of the STARS & R-MARS

This EFA was carried out as per the main EFA in the manuscript, except we only included the STARS and R-MARS and not their modified versions (as per the Phase 1A analysis in our preregistration). There was some discrepancy between the indicators (Table D1), but we retained the seven-factor solution because even though the modal number of factors was eight, there was very little difference between the seven- and eight-factor values for both the RMSEA and SRMR indicators and because parallel analysis is generally the most accurate (Courtney and Gordon 2013; Ruscio and Roche 2012).

## **Table D1**

*The Number of Factors to Extract According to each Indicator in the Phase 1A Polychoric EFA.*

| Indicator | SABIC | RMSEA | SRMR | PA | BIC | Scree Plot | MAP | VSS1 | VSS 2 |
| --- | --- | --- | --- | --- | --- | --- | --- | --- | --- |
| No. of Factors | 8 | 8 | 8 | 7 | 7 | 6 | 5 | 1 | 2 |

*Note.* SABIC = Sample-size-adjusted Bayesian information criterion; RMSEA = Root mean squared error of approximation; SRMR = standardised root mean squared residual; BIC = Bayesian information criterion; Velicer MAP = Velicer’s Minimum Average Partial; VSS = Very simple structure.

The factor loadings (Table D2) suggested a Course Anxiety factor with items from both the STARS and R-MARS, but there are others that show specificity. However, the Phase 1B analysis with the modified versions of the STARS and R-MARS demonstrated that this specificity was primarily driven by constructs not specific to statistics and mathematics.

## Table D2

*Results of the Phase 1A Exploratory Factor Analysis Seven-Factor Solution.*

| Item | 1 | 2 | 3 | 4 | 5 | 6 | 7 |
| --- | --- | --- | --- | --- | --- | --- | --- |
| **Factor 1: Interpretation Anxiety** | | | | | | | |
| STARS Int: Trying to understand the statistical analyses described in the abstract of a journal article | **0.83** | -0.07 | -0.04 | 0.07 | 0.06 | 0.09 | -0.02 |
| STARS Int: Reading a journal article that includes some statistical analyses | **0.77** | 0.05 | 0.03 | -0.09 | 0.07 | 0.05 | 0.00 |
| STARS Int: Interpreting the meaning of a table in a journal article | **0.77** | 0.00 | 0.01 | -0.08 | 0.00 | 0.08 | 0.07 |
| STARS Int: Interpreting the meaning of a probability value once I have found it | **0.66** | 0.19 | -0.01 | 0.03 | -0.04 | -0.04 | 0.11 |
| STARS Int: Trying to understand the odds in a lottery | **0.61** | 0.12 | 0.01 | 0.02 | -0.02 | 0.00 | -0.10 |
| STARS Int: Making an objective decision based on empirical data | **0.60** | 0.21 | -0.02 | 0.01 | -0.08 | 0.06 | 0.18 |
| STARS Int: Watching a student search through a load of computer output from his/her research | **0.60** | 0.01 | 0.11 | -0.01 | 0.09 | 0.09 | -0.02 |
| STARS Int: Trying to decide which analysis is appropriate for my research project | **0.57** | -0.09 | 0.12 | 0.06 | -0.05 | 0.04 | 0.23 |
| STARS Int: Reading an advertisement for a car which includes figures on miles per gallon, depreciation, etc. | **0.53** | 0.18 | 0.11 | -0.04 | 0.13 | -0.10 | -0.17 |
| STARS Int: Determining whether to reject or retain the null hypothesis | **0.53** | 0.11 | 0.06 | 0.11 | -0.03 | 0.01 | 0.04 |
| STARS Int: Arranging to have a body of data put into the computer | **0.49** | 0.07 | 0.20 | -0.02 | 0.01 | 0.19 | 0.06 |
| STARS Test: Finding that another student in class got a different answer than I did to a statistical problem | **0.39** | -0.11 | 0.27 | 0.23 | 0.14 | -0.09 | 0.08 |
| STARS Test: Going over a final examination in statistics after it has been marked | **0.33** | -0.03 | 0.19 | 0.23 | 0.05 | -0.01 | 0.15 |
| **Factor 2: Numeric Task Anxiety** | | | | | | | |
| R-MARS Num: Being given a set of subtraction problems to solve | -0.09 | **0.94** | 0.04 | -0.02 | -0.01 | 0.05 | 0.02 |
| R-MARS Num: Being given a set of division problems to solve | 0.06 | **0.80** | -0.03 | -0.02 | 0.10 | -0.02 | 0.10 |
| R-MARS Num: Being given a set of multiplication problems to solve | 0.13 | **0.75** | -0.06 | 0.16 | 0.03 | -0.02 | -0.04 |
| R-MARS Num: Being given a set of numerical problems involving addition to solve on paper | 0.06 | **0.70** | -0.01 | 0.10 | 0.05 | 0.08 | -0.02 |
| R-MARS Num: Reading a cash register receipt after your purchase | 0.26 | **0.52** | 0.17 | -0.11 | -0.06 | -0.05 | -0.10 |
| **Factor 3: Fear of Asking for Help** | | | | | | | |
| STARS Help: Asking one of my teachers for help in understanding statistical output | -0.06 | 0.01 | **0.96** | -0.02 | 0.02 | -0.01 | 0.01 |
| STARS Help: Going to ask my statistics teacher for individual help with material I am having difficulty understanding | -0.12 | 0.01 | **0.93** | -0.01 | 0.03 | 0.04 | 0.05 |
| STARS Help: Asking someone in the computer lab for help in understanding statistical output | 0.12 | -0.07 | **0.87** | 0.02 | 0.03 | -0.03 | -0.05 |
| STARS Help: Asking a fellow student for help in understanding output | 0.12 | 0.04 | **0.80** | 0.00 | -0.12 | 0.00 | 0.02 |
| **Factor 4: Mathematics Test Result Anxiety** | | | | | | | |
| R-MARS Test: Receiving your final maths grade | -0.04 | 0.07 | 0.11 | **0.70** | -0.02 | 0.02 | 0.10 |
| R-MARS Test: Being given a suprise test in a maths class | 0.07 | 0.05 | 0.02 | **0.66** | 0.12 | 0.08 | -0.01 |
| R-MARS Test: Taking an exam (final) in a maths course | -0.07 | 0.03 | -0.04 | **0.64** | 0.21 | 0.07 | 0.17 |
| R-MARS Test: Taking an exam (quiz) in a maths course | 0.02 | 0.19 | -0.01 | **0.56** | 0.22 | 0.07 | 0.03 |
| R-MARS Test: Taking the maths section of a university entrance exam | -0.01 | 0.11 | 0.04 | **0.53** | 0.11 | 0.14 | 0.08 |
| **Factor 5: Anticipatory Mathematics Tests Anxiety** | | | | | | | |
| R-MARS Test: Thinking about an upcoming maths test 1 day before | 0.02 | 0.02 | 0.03 | 0.14 | **0.78** | 0.03 | 0.03 |
| R-MARS Test: Thinking about an upcoming maths test 1 week before | 0.03 | 0.10 | 0.02 | -0.04 | **0.74** | 0.12 | 0.01 |
| R-MARS Test: Thinking about an upcoming maths test 1 hour before | 0.02 | 0.01 | 0.02 | **0.40** | **0.54** | -0.02 | 0.05 |
| **Factor 6: Course Anxiety** | | | | | | | |
| R-MARS Course: Signing up for a maths course | 0.02 | 0.14 | 0.07 | 0.09 | 0.13 | **0.64** | -0.10 |
| STARS Test: Enrolling in a statistics course | 0.20 | -0.01 | 0.04 | -0.03 | 0.02 | **0.63** | 0.22 |
| R-MARS Test: Realizing you have to take a certain number of maths classes to fulfill requirements for your degree | 0.10 | 0.10 | 0.01 | **0.31** | 0.03 | **0.54** | -0.09 |
| R-MARS Test: Studying for a maths test | 0.02 | 0.13 | 0.03 | 0.18 | **0.32** | **0.38** | 0.04 |
| STARS Test: Doing the coursework for a statistics course | 0.24 | -0.10 | 0.13 | 0.02 | 0.10 | **0.35** | **0.33** |
| **Factor 7: Statistics Test Anxiety** |  |  |  |  |  |  |  |
| STARS Test: Doing an examination in a statistics course | 0.11 | 0.10 | 0.12 | 0.30 | 0.00 | 0.04 | **0.62** |
| STARS Test: Walking into the room to take a statistics test | 0.13 | -0.04 | 0.07 | 0.22 | 0.15 | 0.03 | **0.52** |
| STARS Test: Waking up in the morning on the day of a statistics test | 0.11 | 0.09 | 0.06 | 0.06 | **0.42** | -0.07 | **0.51** |
| STARS Test: Studying for an examination in a statistics course | 0.11 | 0.05 | 0.07 | -0.10 | 0.20 | **0.36** | **0.43** |
| **Stray Items** |  |  |  |  |  |  |  |
| R-MARS Course: Walking into a maths class | 0.04 | 0.27 | 0.27 | 0.16 | 0.09 | 0.26 | -0.09 |
| R-MARS Course: Listening to another student explain a maths formula | 0.22 | 0.25 | 0.19 | 0.10 | 0.09 | 0.25 | -0.12 |
| R-MARS Course: Watching a teacher work on an algebraic equation on the board | 0.24 | 0.29 | 0.09 | 0.22 | -0.01 | 0.27 | -0.15 |
| R-MARS Course: Buying a maths textbook | 0.11 | 0.28 | 0.24 | -0.07 | 0.11 | 0.27 | -0.17 |

*Note.* STARS Int = STARS Interpretation Anxiety subscale item; STARS Help = STARS Fear of Asking for Help subscale item; STARS Test = STARS Test and Class Anxiety subscale item; R-MARS Test = R-MARS Mathematics Test Anxiety subscale item; R-MARS Course = R-MARS Course Anxiety subscale item; R-MARS Num = R-MARS Numerical Task Anxiety subscale item; Bold = factor loadings > |.03|.

# Section E: Latent Profile Analysis

## **Table E1**

*Model Statistics for k = 1:6 Profile Solutions in the Study 1 Sample.*

| Profiles | AIC | BIC | SABIC | Entropy | Probability (Min) | Probability (Max) | *N* (Min) | *N* (Max) | BLRT | BLRT *p* |
| --- | --- | --- | --- | --- | --- | --- | --- | --- | --- | --- |
| 1.00 | 5,562.88 | 5,596.42 | 5,571.03 | 1.00 | 1.00 | 1.00 | 1.00 | 1.00 | NA | NA |
| 2.00 | 4,588.94 | 4,643.44 | 4,602.17 | .86 | .96 | .96 | 0.49 | 0.51 | 983.95 | .01 |
| 3.00 | 4,123.09 | 4,198.55 | 4,141.42 | .87 | .93 | .95 | 0.24 | 0.49 | 475.84 | .01 |
| 4.00 | 3,919.32 | 4,015.75 | 3,942.75 | .86 | .91 | .95 | 0.16 | 0.35 | 213.77 | .01 |
| 5.00 | 3,868.63 | 3,986.01 | 3,897.14 | .89 | .85 | .96 | 0.02 | 0.33 | 60.70 | .01 |
| 6.00 | 3,771.34 | 3,909.69 | 3,804.94 | .87 | .87 | .94 | 0.02 | 0.29 | 107.29 | .01 |

*Note.* AIC = Akaike Information Criterion; BIC = Bayesian Information Criterion; SABIC = Sample Size Adjusted Bayesian Information Criterion; Probability (Min) = the minimum posterior probability of an individual being assigned to a specific profile; Probability (Max) = the maximum posterior probability of an individual being assigned to a specific profile; *N* (Min) = estimated minimum proportion of individuals in each profile; *N* (Max) = estimated maximum proportion of individuals in each profile; BLRT = Bootstrapped Likelihood Ratio Test; BLRT *p* = *p*-value for the BLRT.

## **Table E2**

*Model Statistics for the k = 6 Profile Solution for Variance/Covariance Models 1, 2, 3, and 4 in the Study 1 Sample.*

| Model | AIC | BIC | SABIC | Entropy | Probability (Min) | Probability (Max) | *N* (Min) | *N* (Max) | BLRT | BLRT *p* |
| --- | --- | --- | --- | --- | --- | --- | --- | --- | --- | --- |
| 1.00 | 3,771.34 | 3,909.69 | 3,804.94 | .87 | .87 | .94 | 0.02 | 0.29 | 107.29 | .01 |
| 2.00 | 3,763.95 | 3,986.15 | 3,817.93 | .86 | .76 | .95 | 0.05 | 0.25 | 116.67 | .01 |
| 3.00 | 3,506.34 | 3,669.84 | 3,546.06 | .68 | .64 | .85 | 0.02 | 0.37 | 17.98 | .04 |
| 4.00 | 3,414.95 | 3,788.07 | 3,505.59 | .76 | .68 | .94 | 0.05 | 0.49 | 62.83 | .04 |

*Note.* AIC = Akaike Information Criterion; BIC = Bayesian Information Criterion; SABIC = Sample Size Adjusted Bayesian Information Criterion; Probability (Min) = the minimum posterior probability of an individual being assigned to a specific profile; Probability (Max) = the maximum posterior probability of an individual being assigned to a specific profile; *N* (Min) = estimated minimum proportion of individuals in each profile; *N* (Max) = estimated maximum proportion of individuals in each profile; BLRT = Bootstrapped Likelihood Ratio Test; BLRT *p* = *p*-value for the BLRT.

## **Table E3**

*Model Statistics for k = 1:6 Profile Solutions in the Study 2 Sample.*

| Profiles | AIC | BIC | SABIC | Entropy | Probability (Min) | Probability (Max) | *N* (Min) | *N* (Max) | BLRT | BLRT *p* |
| --- | --- | --- | --- | --- | --- | --- | --- | --- | --- | --- |
| 1.00 | 2,793.11 | 2,821.12 | 2,795.76 | 1.00 | 1.00 | 1.00 | 1.00 | 1.00 | NA | NA |
| 2.00 | 2,357.56 | 2,403.08 | 2,361.87 | 0.85 | 0.95 | 0.97 | 0.44 | 0.56 | 445.55 | 0.01 |
| 3.00 | 2,153.04 | 2,216.07 | 2,159.01 | 0.87 | 0.91 | 0.95 | 0.23 | 0.50 | 214.52 | 0.01 |
| 4.00 | 2,051.81 | 2,132.34 | 2,059.43 | 0.88 | 0.91 | 0.97 | 0.11 | 0.38 | 111.23 | 0.01 |
| 5.00 | 2,010.65 | 2,108.68 | 2,019.93 | 0.90 | 0.92 | 0.96 | 0.03 | 0.38 | 51.16 | 0.01 |
| 6.00 | 1,984.27 | 2,099.81 | 1,995.20 | 0.88 | 0.86 | 0.97 | 0.03 | 0.33 | 36.38 | 0.01 |

*Note.* AIC = Akaike Information Criterion; BIC = Bayesian Information Criterion; SABIC = Sample Size Adjusted Bayesian Information Criterion; Probability (Min) = the minimum posterior probability of an individual being assigned to a specific profile; Probability (Max) = the maximum posterior probability of an individual being assigned to a specific profile; *N* (Min) = estimated minimum proportion of individuals in each profile; *N* (Max) = estimated maximum proportion of individuals in each profile; BLRT = Bootstrapped Likelihood Ratio Test; BLRT *p* = *p*-value for the BLRT.

## Table E4

*Model Statistics for the k = 6 Profile Solution for Variance/Covariance Models 1, 2, 3, and 4 in the Study 2 Sample.*

| Model | AIC | BIC | SABIC | Entropy | Probability (Min) | Probability (Max) | *N* (Min) | *N* (Max) | BLRT | BLRT p |
| --- | --- | --- | --- | --- | --- | --- | --- | --- | --- | --- |
| 1.00 | 1,984.27 | 2,099.81 | 1,995.20 | 0.88 | 0.86 | 0.97 | 0.03 | 0.33 | 36.38 | 0.01 |
| 2.00 | 1,987.78 | 2,173.35 | 2,005.34 | 0.91 | 0.90 | 0.98 | 0.04 | 0.34 | 31.89 | 0.01 |
| 3.00 | 1,871.23 | 2,007.77 | 1,884.15 | 0.71 | 0.63 | 0.99 | 0.03 | 0.36 | 20.13 | 0.05 |
| 4.00 | 1,837.73 | 2,149.34 | 1,867.22 | 0.81 | 0.73 | 1.00 | 0.03 | 0.32 | 48.94 | 0.14 |

*Note.* AIC = Akaike Information Criterion; BIC = Bayesian Information Criterion; SABIC = Sample Size Adjusted Bayesian Information Criterion; Probability (Min) = the minimum posterior probability of an individual being assigned to a specific profile; Probability (Max) = the maximum posterior probability of an individual being assigned to a specific profile; *N* (Min) = estimated minimum proportion of individuals in each profile; *N* (Max) = estimated maximum proportion of individuals in each profile; BLRT = Bootstrapped Likelihood Ratio Test; BLRT *p* = *p*-value for the BLRT.

## Table E5

*Profile Means (Centred) and Frequencies for the Six Profiles in Study 1.*

| Profile | STARS | STARS-M | R-MARS | R-MARS-S | *n* | % |
| --- | --- | --- | --- | --- | --- | --- |
| 1 | 0.80 | 0.87 | 0.83 | 0.80 | 109 | 22.3 |
| 2 | -1.46 | -1.37 | -1.33 | -1.54 | 70 | 14.3 |
| 3 | 1.69 | 1.78 | 1.75 | 1.61 | 40 | 8.2 |
| 4 | -0.68 | -0.64 | -0.51 | -0.59 | 122 | 25.0 |
| 5 | 0.52 | -0.49 | -0.95 | 0.34 | 26 | 5.3 |
| 6 | 0.11 | 0.16 | 0.16 | 0.14 | 122 | 25.0 |

## Table E6

*Profile Means (Centred) and Frequencies for the Six Profiles in Study 2.*

| Profile | STARS | STARS-M | R-MARS | R-MARS-S | n | % |
| --- | --- | --- | --- | --- | --- | --- |
| 1 | 0.01 | 0.30 | 0.69 | 0.06 | 30 | 12.2 |
| 2 | –1.06 | –0.84 | –0.69 | –0.94 | 68 | 27.8 |
| 3 | 0.20 | 0.45 | 0.39 | 0.25 | 29 | 11.8 |
| 4 | 0.44 | 0.13 | –0.14 | 0.21 | 79 | 32.2 |
| 5 | 0.41 | –1.29 | –1.75 | 0.58 | 8 | 3.3 |
| 6 | 1.15 | 1.36 | 1.44 | 1.33 | 31 | 12.7 |

# Section F: Multi-Level Models

## Table F1

*Descriptive Statistics for all Variables in the Multilevel Models for Study 1, Split by MCQ Condition (Statistics or Mathematics).*

|  |  |  |  |  |  |  |  | 95% CI | |
| --- | --- | --- | --- | --- | --- | --- | --- | --- | --- |
| MCQ | Variable | *M* | *SD* | Min | Max | Skew | Kurtosis | Lower | Upper |
| Mathematics | R-MARS | 2.77 | .83 | 1.00 | 4.85 | 0.31 | 2.69 | 2.66 | 2.88 |
| Statistics | R-MARS | 2.79 | .74 | 1.00 | 4.80 | 0.15 | 2.59 | 2.69 | 2.88 |
| Mathematics | STARS | 2.91 | .82 | 1.04 | 4.87 | 0.13 | 2.47 | 2.80 | 3.01 |
| Statistics | STARS | 2.95 | .76 | 1.17 | 4.91 | 0.02 | 2.59 | 2.86 | 3.05 |
| Mathematics | State Anxiety (In-Test) | 1.77 | .62 | 1.00 | 3.76 | 0.84 | 3.04 | 1.69 | 1.85 |
| Statistics | State Anxiety (In-Test) | 1.79 | .67 | 1.00 | 3.95 | 0.96 | 3.15 | 1.71 | 1.88 |
| Mathematics | State Anxiety (Pre-Test) | 1.73 | .59 | 1.00 | 3.52 | 0.80 | 2.87 | 1.66 | 1.81 |
| Statistics | State Anxiety (Pre-Test) | 1.82 | .63 | 1.00 | 3.71 | 0.79 | 3.19 | 1.74 | 1.90 |
| Mathematics | Trait Anxiety | 2.17 | .64 | 1.00 | 3.90 | 0.33 | 2.53 | 2.09 | 2.25 |
| Statistics | Trait Anxiety | 2.25 | .62 | 1.00 | 3.71 | 0.14 | 2.45 | 2.18 | 2.33 |

*Note. M* = mean; *SD* = standard deviation; Min = minimum recorded value; Max = maximum recorded value; 95% CI = 95% confidence interval; Lower = Lower bound of the 95% confidence interval; Upper = Upper bound of the 95% confidence interval.

## Table F2

*Descriptive Statistics for all Variables in the Multilevel Models for Study 2, Split by MCQ Condition (Statistics or Mathematics).*

|  |  |  |  |  |  |  |  | 95% CI | |
| --- | --- | --- | --- | --- | --- | --- | --- | --- | --- |
| MCQ | Variable | *M* | *SD* | Min | Max | Skew | Kurtosis | Lower | Upper |
| Mathematics | R-MARS | 3.03 | .79 | 1.20 | 4.60 | -0.16 | 2.34 | 2.89 | 3.17 |
| Statistics | R-MARS | 3.08 | .78 | 1.20 | 4.70 | -0.14 | 2.48 | 2.94 | 3.22 |
| Mathematics | STARS | 3.26 | .75 | 1.61 | 4.96 | -0.10 | 2.45 | 3.12 | 3.39 |
| Statistics | STARS | 3.23 | .75 | 1.43 | 4.74 | -0.16 | 2.50 | 3.09 | 3.36 |
| Mathematics | State Anxiety (In-Test) | 2.08 | .72 | 1.00 | 4.00 | 0.38 | 2.35 | 1.95 | 2.21 |
| Statistics | State Anxiety (In-Test) | 2.12 | .66 | 1.00 | 3.90 | 0.36 | 2.57 | 2.00 | 2.24 |
| Mathematics | State Anxiety (Pre-Test) | 1.98 | .64 | 1.00 | 3.43 | 0.33 | 2.13 | 1.86 | 2.09 |
| Statistics | State Anxiety (Pre-Test) | 1.95 | .60 | 1.00 | 3.71 | 0.33 | 2.50 | 1.84 | 2.05 |
| Mathematics | Trait Anxiety | 2.52 | .67 | 1.00 | 4.00 | -0.11 | 2.40 | 2.40 | 2.64 |
| Statistics | Trait Anxiety | 2.46 | .60 | 1.24 | 3.86 | -0.14 | 2.44 | 2.36 | 2.57 |

*Note. M* = mean; *SD* = standard deviation; Min = minimum recorded value; Max = maximum recorded value; 95% CI = 95% confidence interval; Lower = Lower bound of the 95% confidence interval; Upper = Upper bound of the 95% confidence interval.

## Table F3

*Multi-Level Model Results from Study 1, Model 1.*

| Parameter | $\hat{\beta}$ | 95% CI | $t$ | $df$ | $p$ |
| --- | --- | --- | --- | --- | --- |
| Intercept | .28 | [0.14, 0.42] | 3.82 | 488.23 | < .001 |
| Trait Anxiety | .67 | [0.61, 0.73] | 21.62 | 474.00 | < .001 |
| Time (Pre-Test/In-Test) | .04 | [-0.01, 0.09] | 1.49 | 474.00 | .137 |
| MCQ (Statistics) | .03 | [-0.05, 0.10] | 0.71 | 472.53 | .475 |
| STARS | .04 | [-0.02, 0.09] | 1.33 | 488.81 | .184 |
| Time $\times$ MCQ (Statistics) | -.07 | [-0.14, 0.00] | -1.86 | 474.00 | .063 |
| Time $\times$ STARS | .08 | [0.03, 0.13] | 3.10 | 474.00 | .002 |
| MCQ (Statistics) $\times$ STARS | .03 | [-0.04, 0.10] | 0.76 | 472.22 | .446 |
| Time $\times$ MCQ (Statistics) $\times$ STARS | -.04 | [-0.11, 0.04] | -0.99 | 474.00 | .321 |

*Note.* $\hat{\beta}$ = standardised beta estimate; 95% CI = 95% confidence interval; *t* = t-statistic; *df* = degrees of freedom; *p* = *p*-value.

## Table F4

*Multi-Level Model Results from Study 1, Model 2.*

| Parameter | $\hat{\beta}$ | 95% CI | $t$ | $df$ | $p$ |
| --- | --- | --- | --- | --- | --- |
| Intercept | .24 | [0.10, 0.37] | 3.42 | 489.12 | < .001 |
| Trait Anxiety | .69 | [0.63, 0.75] | 23.37 | 474.00 | < .001 |
| Time (Pre-Test/In-Test) | .04 | [-0.01, 0.09] | 1.44 | 474.00 | .151 |
| MCQ (Statistics) | .03 | [-0.04, 0.10] | 0.73 | 472.91 | .463 |
| R-MARS | .01 | [-0.04, 0.06] | 0.29 | 482.14 | .769 |
| Time × MCQ (Mathematics) | -.07 | [-0.14, 0.01] | -1.82 | 474.00 | .070 |
| Time × R-MARS | .08 | [0.04, 0.13] | 3.44 | 474.00 | < .001 |
| MCQ (Mathematics) × R-MARS | .05 | [-0.02, 0.12] | 1.41 | 472.39 | .160 |
| Time × MCQ (Mathematics) × R-MARSS | -.02 | [-0.10, 0.05] | -0.61 | 474.00 | .542 |

*Note.* $\hat{\beta}$ = standardised beta estimate; 95% CI = 95% confidence interval; *t* = t-statistic; *df* = degrees of freedom; *p* = *p*-value.

## Figure F1

*Predicted State Anxiety Change at Different Levels of Statistics Anxiety for each MCQ Condition in the Study 1 Sample.*

*Note.* Statistics Anxiety = -1 = 1 standard deviation below the mean; Statistics Anxiety = 0 = the mean; Statistics Anxiety = +1 = 1 standard deviation above the mean.

## Figure F2

*Predicted State Anxiety Change at Different Levels of Mathematics Anxiety for each MCQ Condition in the Study 1 Sample.*

*Note.* Mathematics Anxiety = -1 = 1 standard deviation below the mean; Mathematics Anxiety = 0 = the mean; Mathematics Anxiety = +1 = 1 standard deviation above the mean.

## Table F5

*Multi-Level Model Results from Study 2, Model 1.*

| Predictor | $\hat{\beta}$ | 95% CI | $t$ | $df$ | $p$ |
| --- | --- | --- | --- | --- | --- |
| Intercept | .32 | [0.09, 0.56] | 2.70 | 254.22 | .007 |
| Trait Anxiety | .66 | [0.57, 0.74] | 14.65 | 242.00 | < .001 |
| Time (Pre-Test/In-Test) | .10 | [0.02, 0.19] | 2.50 | 242.00 | .013 |
| MCQ (Statistics) | .01 | [-0.10, 0.12] | 0.16 | 242.07 | .873 |
| STARS | .03 | [-0.06, 0.12] | 0.71 | 257.54 | .481 |
| Time $\times$ MCQ (Statistics) | .07 | [-0.05, 0.19] | 1.17 | 242.00 | .241 |
| Time $\times$ STARS | .14 | [0.06, 0.22] | 3.37 | 242.00 | < .001 |
| MCQ (Statistics) $\times$ STARS | .00 | [-0.11, 0.11] | 0.00 | 244.24 | .999 |
| Time $\times$ MCQ (Statistics) $\times$ STARS | -.03 | [-0.15, 0.09] | -0.50 | 242.00 | .621 |

*Note.* $\hat{\beta}$ = standardised beta estimate; 95% CI = 95% confidence interval; *t* = t-statistic; *df* = degrees of freedom; *p* = *p*-value.

## Table F6

*Multi-Level Model Results from Study 2, Model 2.*

| Predictor | $\hat{\beta}$ | 95% CI | $t$ | $df$ | $p$ |
| --- | --- | --- | --- | --- | --- |
| Intercept | .29 | [0.05, 0.52] | 2.39 | 253.65 | .018 |
| Trait Anxiety | .67 | [0.58, 0.76] | 15.08 | 242 | < .001 |
| Time (Pre-Test/In-Test) | .11 | [0.03, 0.19] | 2.62 | 242 | .009 |
| MCQ (Mathematics) | .01 | [-0.11, 0.12] | 0.13 | 242.07 | .899 |
| R-MARS | .02 | [-0.06, 0.10] | 0.47 | 255.51 | .636 |
| Time × MCQ (Mathematics) | .06 | [-0.06, 0.18] | 0.97 | 242 | .331 |
| Time × R-MARS | .13 | [0.04, 0.21] | 2.99 | 242 | .003 |
| MCQ (Mathematics) × R-MARS | .01 | [-0.11, 0.12] | 0.14 | 243.68 | .886 |
| Time × MCQ (Mathematics) × R-MARS | -.05 | [-0.17, 0.07] | -0.88 | 242 | .382 |

*Note.* $\hat{\beta}$ = standardised beta estimate; 95% CI = 95% confidence interval; *t* = t-statistic; *df* = degrees of freedom; *p* = *p*-value.

## Figure F3

*Predicted State Anxiety Change at Different Levels of Statistics Anxiety for each MCQ Condition in the Study 2 Sample.*

*Note.* Statistics Anxiety = -1 = 1 standard deviation below the mean; Statistics Anxiety = 0 = the mean; Statistics Anxiety = +1 = 1 standard deviation above the mean.

## Figure F4

*Predicted State Anxiety Change at Different Levels of Mathematics Anxiety for each MCQ Condition in the Study 2 Sample.*

*Note.* Mathematics Anxiety = -1 = 1 standard deviation below the mean; Mathematics Anxiety = 0 = the mean; Mathematics Anxiety = +1 = 1 standard deviation above the mean.
